# Orthologs of NOX5 and EC-SOD/SOD3: dNox and dSod3 Impact Egg Hardening Process and Egg Laying in Reproductive Function of *Drosophila melanogaster*

**DOI:** 10.3390/ijms25116138

**Published:** 2024-06-02

**Authors:** Eva Louise Steinmetz, Annika Scherer, Célestine Calvet, Uli Müller

**Affiliations:** Zoology & Physiology, ZHMB (Center of Human and Molecular Biology), Saarland University, Building B2.1, D-66123 Saarbrücken, Germany

**Keywords:** NADPH oxidase 5 (NOX5), (extracellular) superoxide dismutase (EC-SOD or SOD3)

## Abstract

The occurrence of ovarian dysfunction is often due to the imbalance between the formation of reactive oxygen species (ROS) and the ineffectiveness of the antioxidative defense mechanisms. Primary sources of ROS are respiratory electron transfer and the activity of NADPH oxidases (NOX) while superoxide dismutases (SOD) are the main key regulators that control the levels of ROS and reactive nitrogen species intra- and extracellularly. Because of their central role SODs are the subject of research on human ovarian dysfunction but sample acquisition is low. The high degree of cellular and molecular similarity between *Drosophila melanogaster* ovaries and human ovaries provides this model organism with the best conditions for analyzing the role of ROS during ovarian function. In this study we clarify the localization of the ROS-producing enzyme dNox within the ovaries of *Drosophila melanogaster* and by a tissue-specific knockdown we show that dNox-derived ROS are involved in the chorion hardening process. Furthermore, we analyze the dSod3 localization and show that reduced activity of dSod3 impacts egg-laying behavior but not the chorion hardening process.

## 1. Introduction

Reactive oxygen species (ROS) is used as a collective term for byproducts that arise from the incomplete reduction of molecular oxygen (O_2_) during normal aerobic metabolism. Free radicals, such as hydroxyl (OH^●^) and superoxide anion (O_2_^●−^), are highly reactive and can cause damage to proteins, DNA, and lipids. Therefore, it is essential to keep the formation and elimination of ROS under control to maintain physiological metabolic functions. Antioxidative scavenging mechanisms counteract the production of ROS to prevent excessive ROS production.

On the other hand, superoxide anions and non-radical hydrogen peroxide (H_2_O_2_) can act as signaling molecules or effectors within and between cells [1,2,3,4]. In the female reproductive system, ROS and antioxidants are considered key factors in the physiological metabolism of the ovary, as they have been shown to influence the reproductive processes of female mammals, such as folliculogenesis, ovulation, fertilization, corpus luteum maintenance/regression and embryogenesis [5,6,7,8,9].

Sources of ROS in the ovary can be macrophages and neutrophils, steroidogenic cells, and intracellular sites of origin such as mitochondria, endoplasmic reticulum, nuclear membrane electron transport, and plasma membranes (as reviewed in [5]). To maintain the delicate redox balance, an antioxidant defense system composed of enzymatic and non-enzymatic antioxidants ensures ovarian homeostasis (reviewed in [8]). Enzymatic antioxidants such as superoxide dismutase (SOD), catalase, glutathione peroxidase, glutathione reductase, and glutathione oxidase are abundant in regions of ROS production within a cell. The most harmful ROS, the superoxide anion, is removed by SOD in a dismutation reaction. However, SODs not only act as antioxidants that protect the cell from oxidative damage caused by ROS, they can also be considered regulators that can convert one oxidant into another type of oxidant, thereby potentially affecting redox-dependent signaling pathways [10].

The SOD protein family in mammals consists of three members (SOD1–3) encoded by different genes. Exhibiting functional similarities, all forms of SOD catalyze the dismutation of superoxide to hydrogen peroxide but differ in subcellular compartmentalization and cofactor requirements [11,12], and they require catalytic metals for their activation: the cytoplasmic Cu/Zn-SOD (SOD1), the mitochondrial Mn-SOD (SOD2), and the extracellular Cu/Zn-SOD (SOD3 or EC-SOD) [12,13,14]. Being secreted extracellularly, SOD3 is the only enzyme able to remove superoxide from the extracellular space, protecting constituents in this compartment from oxidative modifications. Otherwise, the extracellular activity of SOD3 can be seen as a source of hydrogen peroxide for potential autocrine or paracrine redox signaling. 

As SOD abundance and activity change with female age and in ovarian dysfunctions such as endometriosis and polycystic ovary disease (PCOS) [7,15,16,17], it is under research to become a potential biomarker determining systemic oxidative stress and oocyte quality [15,16]. However, due to limited options for obtaining suitable sample materials, there is a demand for investigations using alternative model organisms. Despite some obvious differences, *Drosophila* and mammalian ovaries have similarities regarding cellular organization and oocyte development, as reviewed in [18]. Concerning both organisms, conserved biological functions, including intercellular signaling, must occur in the ovaries for functional female gametes to be produced. Therefore, analyzing the physiological regulation of ROS in *Drosophila* ovaries can provide important insights. 

In *Drosophila*, three genes encode dSod (dSod1–3) [19,20]. The cytoplasmatic Cu/Zn-dSod (dSod1) (CG11793) [21], the mitochondrial Mn-dSod (dSod2) (CG8905) [22,23], and the extracellular Cu/Zn-dSod (dSod3/EC-Sod3) (CG9027) [24,25]. dSod3 exists in two isoforms; in addition to the extracellular form, a membrane-associated form is predicted [25].

Few things are known about the function or localization of dSod3. The expression rate of *dsod*3 was previously shown to be higher in female compared with male flies [24,25], and the mRNA signal is elevated in ovaries [26], indicating a possible significant function of dSod3 during ovarian processes. Furthermore, using RNASeq, it has been shown that dSod3 is enriched in late-stage egg chambers [27]. The observation of an overexpressed dSod3-HA construct being secreted into the extracellular space of posterior mature follicle cells led to the assumption of a potential extracellular localization of dSod3 in the ovaries [28]. 

The substrate for extracellular SODs, superoxide anions, is formed enzymatically by NADPH oxidases (Nox). In *Drosophila,* two members of the NOX family of proteins exist: dNox (CG34399) and dDuox (CG3131) [20], while there are seven members in mammals (NOX1–5, DUOX1 and DUOX2) [29,30,31]. The dNox, orthologue to the human NOX5, is a protein with six transmembrane domains that contains an EF-hand-containing calcium-binding domain that mediates activation by cytosolic calcium and a cytoplasmic NADPH-binding site, as reviewed in [32,33].

ROS produced by *Drosophila* dNox have been shown to be essential in the ovulation process to control agonist-induced calcium flux during ovarian smooth muscle contraction [34] and for a successful follicle rupture, as has been shown previously during an ex vivo assay of dissected late-stage egg chambers [28]. According to these studies, dNox primarily produces superoxide anions (O_2_^●−^) in the extracellular space, which dismutate spontaneously or are dismutated enzymatically by extracellular superoxide dismutase (dSod3) [28,34]. Using RNASeq, dNox has been shown to be enriched in late-stage egg chambers and was proven separately in the follicle cells of late-stage egg chambers by RT-PCR [27,28]. However, since neither dNox nor dSod3 antibodies have been available, the localization of these enzymes in the *Drosophila* ovary is still unclear. 

In the current study, we first focused on the localization of dNox and dSod3 in *Drosophila* ovaries using immunofluorescence. We generated a specific dNox antibody and tested an available SOD3 antibody. We showed that dNox and dSod3 both localize at the follicular epithel of mid- to late-stage egg chambers and that dSod3 localizes to ovariole muscle sheaths and oviducts. Then, we analyzed the ovaries in the genetic background of a *dsod3* mutant line and knocked down *dnox* and *dsod3* according to the discovered localization. The knockdown experiments revealed severe defects in egg laying that have not been described previously.

## 2. Results

### 2.1. Localization of dNox and dSod3 in Drosophila Ovaries

**Figure 1:**
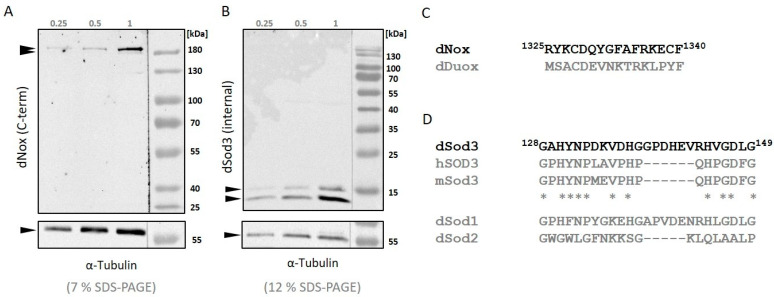
Western blot analysis of whole ovary homogenates with (**A**): anti-dNox antibody (1:500; this work) and (**B**): anti-dSod3 antibody (1:500; #PA5-102904 Invitrogen). The amount of probes is equivalent to 0.25, 0.5, or 1 ovary; α-tubulin was used as a loading control; probes were run on a 7% SDS-PAGE when analyzing dNox and on a 12% SDS-PAGE when analyzing dSod3 (optimized for protein size). (**C**): Alignment of the amino acid sequences that were used as immunogen for the generation of the antibody against Drosophila NADPH oxidase (dNox) (black letters) against the amino acid sequence of the second Drosophila (dual specific) NADPH oxidase (dDuox) (in grey) (**D**): Alignment of the amino acid sequences of Drosophila superoxide dismutase 3 (dSod3) (black letters), human superoxide dismutase 3 (hSOD3), mouse superoxide dismutase 3 (mSod3), and the two remaining Drosophila superoxide dismutase isoforms 1 and 2 (dSod2 and dSod2) (in grey). Asterisks mark amino acid residues that are conserved between dSod3, hSOD3, and mSod3.

The abundance of dNox in Drosophila ovaries has not been tested so far, as a dNox antibody was not available. In order to investigate this task, we generated an antibody against the C-terminal domain of the transmembrane protein dNox (Figure 1A). To avoid a cross-reaction of the antibody with the second member of the Drosophila NADPH oxidase family, the dual-specific NADPH oxidase (dDuox), we ensured that the amino acid sequences of dNox and dDuox were not conserved, especially at the C-terminal end (Figure 1C).

As can be seen in Figure 1, homogenates of wild-type ovaries show a double band, which was also seen with other NOX antibodies in other species [35] and might be representative of the second isoform that is described for dNox.

The localization of dSod3 has so far been described in the extracellular space using the Drosophila SL2 cell line [24] and overexpressing a HA-tagged dSod3 construct secreted into the extracellular space of mature follicle cells [28]. However, due to the lack of a dSod3 antibody suitable for immunohistochemical experiments, the concrete localization of dSod3 has been unknown. In this work, we tested an antibody that was originally made against human superoxide dismutase 3 (hSOD3) to detect Drosophila superoxide dismutase 3 (dSod3). First, we aligned and compared the amino acid sequences of hSOD3, mouse superoxide dismutase (mSod3), and dSod3 (Figure 1D). We found a commercially available antibody against hSOD3 that showed a grade of conservation in the amino acid sequence with dSod3 and, at the same time, low conservation with the remaining isoforms dSod1 and dSod2. Consequently, we decided to test whether we could detect dSod3 specifically (Figure 1B). The two bands of dSod3 detection are a known phenomenon [24]. The upper band nearly reaches the expected molecular weight of about 19 kDa. Further tests for the specifity of both antibodies are shown in Supplementary Materials (Figure S1). All together, we were able to generate an antibody against the C-terminal domain of dNox, and we found an antibody against the catalytic domain of dSod3.

**Figure 2:**
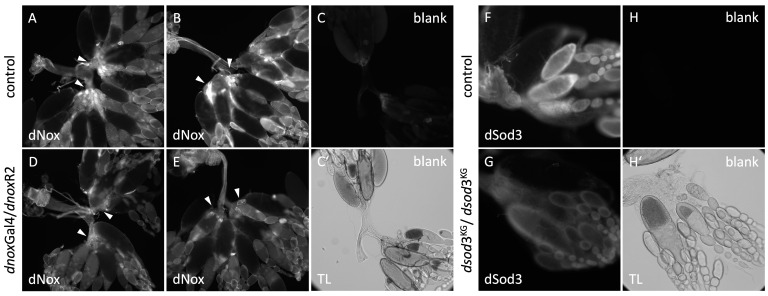
Immunostaining on fixed ovaries with anti-dNox antibody (1:250) and anti-dSod3 antibody (1:250) (**A**,**B**): control ovaries (without RNAi induction; +/UAS*dnox*R2). (**D**,**E**): ovaries with RNAi-downregulated *dnox* expression (*dnox*Gal4(BL78988)/UAS*dnox*R2(BL32902)). Arrowheads point to regions of reduced dNox signal compared with control. (**C**,**C′**): technical control without primary antibody (blank) on control ovaries. TL transmitted light image of the blank. Fluorescence images were taken under identical microscope settings and were processed the same. (**F**): control ovary (+/+) and (**G**): ovary of the homozygous (*dsod3*^KG^/*dsod3*^KG^) mutant. (**H**,**H′**): technical control without primary antibody (blank) on (*+/+*) ovaries. TL transmitted light image of the blank. Fluorescence images were taken under identical microscope settings and were processed the same.

To localize dNox and dSod3 in ovaries, we dissected whole ovaries from adult females and performed indirect immunostaining on fixed tissue (Figure 2).

Immunostaining with the dNox antibody showed signals at different locations of the adult control ovaries and at different stages of egg chamber development (Figure 2A,B). The included technical control (identical immunostaining procedure but without primary antibodies; Figure 2C,C’) confirms that the signal is due to the specific binding of the dNox antibody. Compared with the control ovaries, the signal in ovaries with genetically downregulated dnox is regionally reduced (Figure 2D,E; see arrowheads). As the dnoxGal4 (BL78988) driver line represents a CRIMIC enhancer trap line that probably does not recapitulate the complete endogenous expression pattern of dnox, we used reporter gene expression to visualize the expression that is induced by dnoxGal4 (BL78988) (see Figure S2). The reporter gene expression pattern confirmed dnox gene expression in the oviduct region. Therefore, the reduced dNox antibody signal in the oviduct region, when dnox expression was downregulated by dnox Gal4 (BL78988) (Figure 2D,E, see arrowheads), points towards a specifity of the antibody. Immunostaining with the dSod3 antibody also showed signals at different locations of the adult ovary (Figure 2F). The included technical control (Figure 2G,G’) confirms the signal to be specific from the binding of the dSod3 antibody. Compared with the control ovaries, the signal in homozygous mutant dsod3^KG^ (BL14138) is reduced overall (Figure 2G), pointing towards a specificity of the dSod3 antibody.

**Figure 3:**
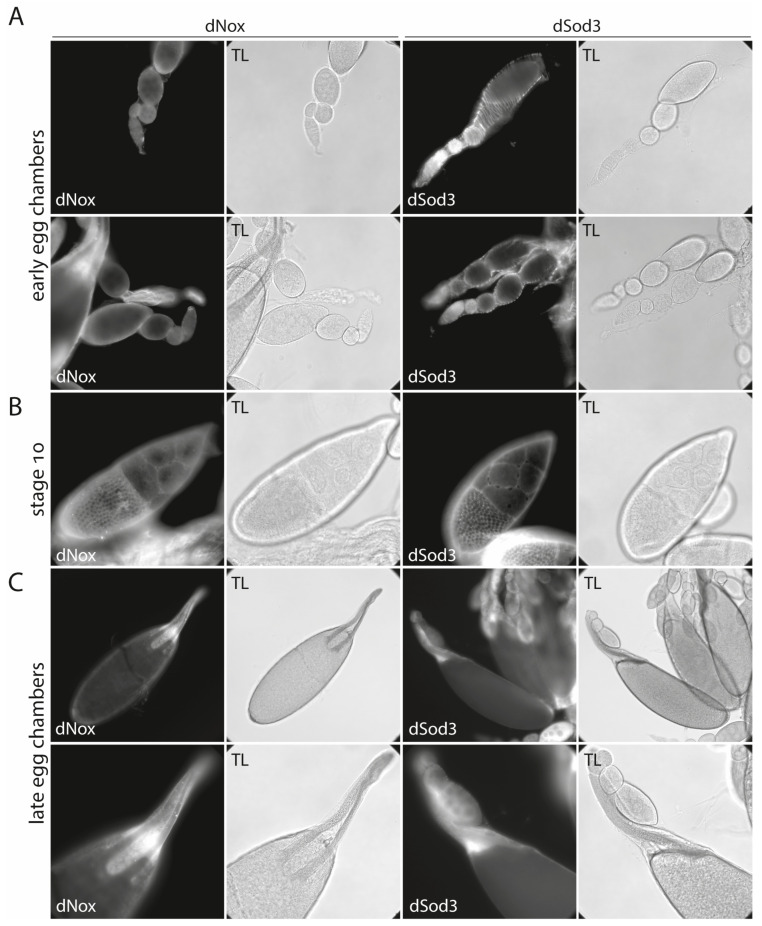
Comparison of dNox and dSod3 localization by immunohistochemistry. (**A**): early egg chambers, (**B**): stage 10 egg chambers, and (**C**): late stages of egg chamber development. (**A**): dNox localization (left panel) and dSod3 localization (right panel) in early egg chambers. (**B**): dNox localization (left panel) and dSod3 localization (right panel) in stage 10 egg chambers. (**C**): dNox localization (left panel) and dSod3 localization (right panel) in the anterior region of late egg chambers. All fluorescent images are accompanied by the respective transmitted light (TL) images for better identification of all structures. Indirect immunostaining was done using anti-dNox (1:250; this work) or anti-dSod3 (1:250; #PA5-102904 Invitrogen) antibodies combined with fluorophore-coupled (Cy3 or Cy5) secondary antibodies on fixed ovaries.

The localization was now checked in all stages of egg chamber development, from germarium to mature follicles, and additionally in the oviduct. The most noticeable and representative regions during egg chamber development are shown in Figure 3.

**Figure 4:**
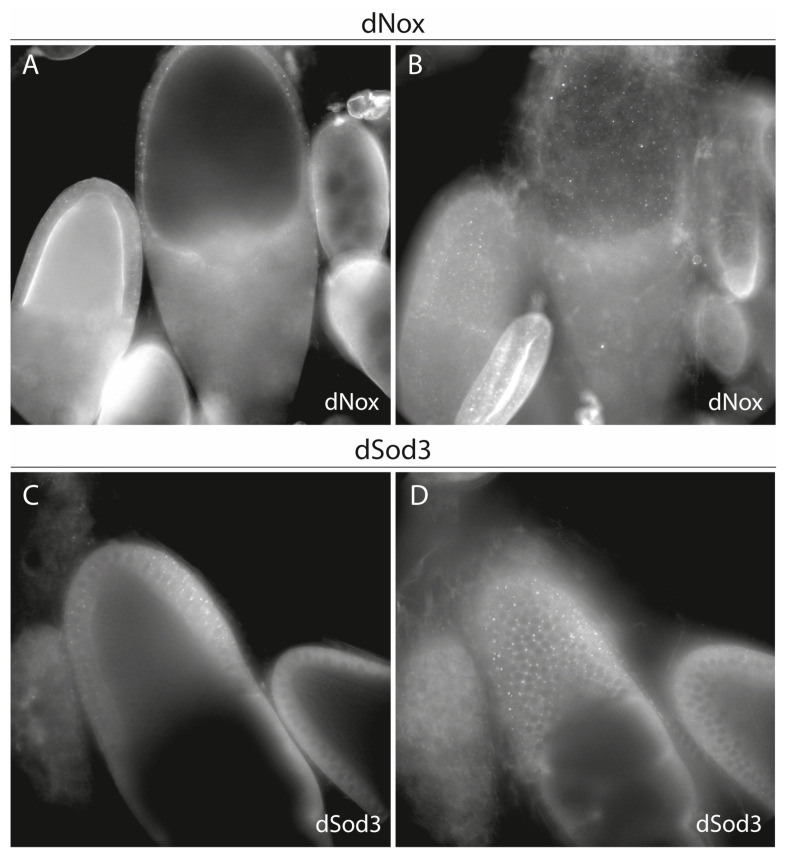
dNox and dSod3 localization in stage 9–10 egg chambers. (**A**,**B**): dNox localization (cross-section focus in (**A**) and surface focus in (**B**) in stage 10 egg chamber and (**C**,**D**): dSod3 localization (cross-section focus in (**C**) and surface focus in (**D**)) in stage 9 egg chamber; focus was set to the follicle cell epithel that surrounds the growing oocyte. Indirect immunostaining was done using anti-dNox (1:250; this work) or anti-dSod3 (1:250; #PA5-102904 Invitrogen) antibodies combined with fluorophore-coupled (Cy3 or Cy5) secondary antibodies on fixed ovaries.

Furthermore, in the ovariole epithelial muscle sheath, both enzymes share a similar protein localization pattern within the middle- to late-stage egg chambers. While dNox shows no specific signal in early egg chambers/ovarioles, dSod3 is expressed in the ovariole muscle sheath that envelops each single ovariole (Figure 3, A left and right panels; see also Figure S3). From the onset of oocyte growth (at egg chamber stage 7/8) and even more prominently at stage 10, dNox and dSod3 are both spatially associated with the tricellular contact regions visible between the main body follicular cells (Figure 3B; see also Figure 4 and Figure S4), which form a monolayer covering the growing oocyte. The distribution of dNox and dSod3 appears in spots in these regions. In late-stage egg chambers, dNox and dSod3 are mainly found at the anterior end of the mature eggs, surrounding the micropyle and operculum area, and, to a much lesser extent, at the follicular epithelium, surrounding the mature egg (stage 14) (Figure 3C). 

As can be seen in this higher magnification, dNox and dSod3 proteins are localized in the follicular epithel covering the oocyte. Regarding the cross sections of the egg chambers (Figure 4A,C), both enzymes appear to be localized on the cell’s surfaces, between the follicle cells and at their apical surfaces toward the oocyte. Focusing on the surface of the egg chambers (Figure 4B,D) supports the observation of localization on the surface/between the epithelial cells. Both proteins are restricted to the follicle cells covering the oocyte/that are in contact with the oocyte.

**Figure 5:**
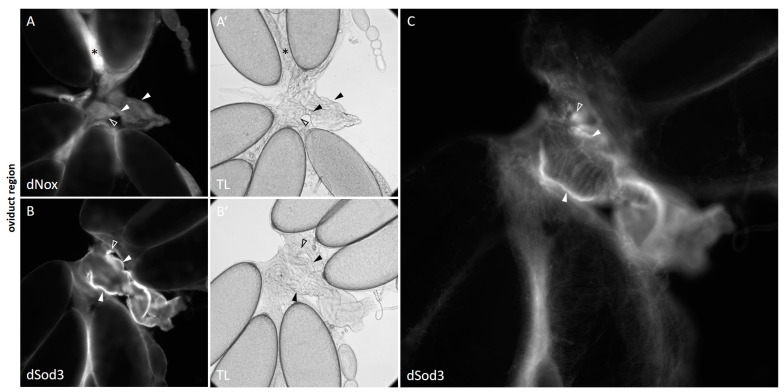
dNox and dSod3 localization in the oviduct. Oviducts of wild-type females were immunostained with: (**A**,**A′**) anti-dNox antibody (1:250; this work) and (**B**,**B′**): anti-dSod3 antibody (1:250; #PA5-102904 Invitrogen) combined with fluorophore-coupled secondary antibodies on fixed ovaries. Fluorescent images are accompanied by the respective transmitted light (TL) images for better identification of all structures. Filled arrowheads point to the common oviduct, and unfilled arrowheads depict parts of the lateral oviducts. While (**A**,**B**) show a cross-section focus, (**C**) shows a magnified and surface-focused view from (**B**) for a more detailed representation of the dSod3 localization in the common oviduct area. Asterisks in (**A**,**A′**) mark a corpus luteum (CL).

As previous studies have shown that the ovulation process depends on proper dNox function [28,34], we also investigated the oviduct region of the ovary for potential dNox and dSod3 presence (Figure 5).

Within the oviduct region of the ovary, dNox was detected in the corpus luteum (CL) and, to a low content, in the common and lateral oviducts (Figure 5A; see also Figures S3A–A″ and S4A–A″). dSod3 is localized in the muscle layer of the common as well as the lateral oviduct (Figure 5B,C; see also Figure S2).

Taken together, both proteins share a similar spatio-temporal distribution pattern in the follicular epithel of developing mid- to late-stage egg chambers. Furthermore, the ovarian muscle tissue appears as a prominent region for dSod3 distribution. We found dSod3 at the epithelial muscle sheath, a thin circular layer that is surrounding every single ovariole, and at the lateral and common oviduct musculature, which represents a circular layer of somatic muscles. 

### 2.2. dNox and dSod3 Are Involved in Egg Laying and/or Egg Hardening Processes

**Figure 6:**
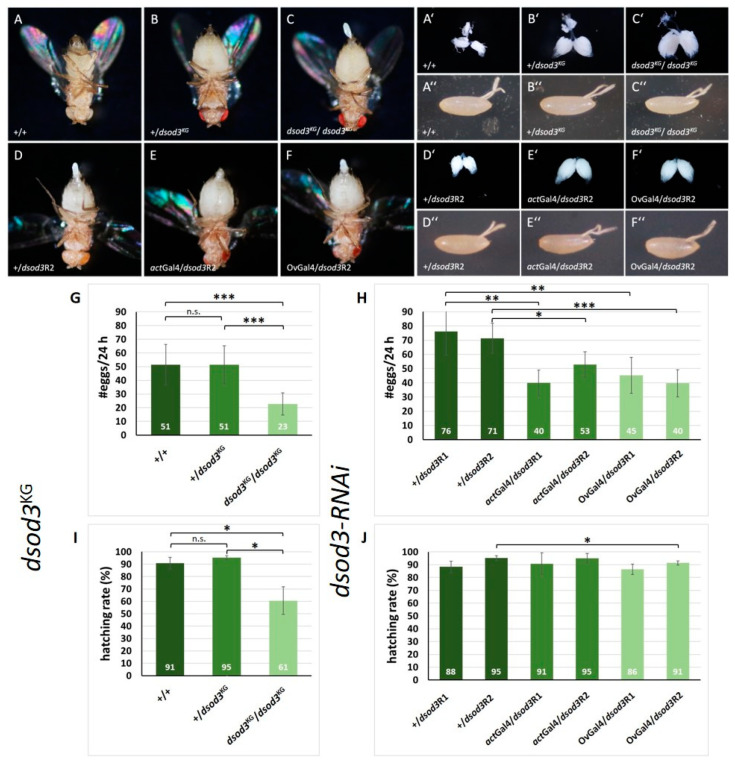
Diminished level of dsod3 has an impact on ovary function and egg-laying. (**A**–**F**): abdominal morphology, (**A′**–**F′**): ovary morphology, and (**A″**–**F″**): laid egg morphology. First lane: control (+/+) compared with females with diminished gene doses of dsod3: heterozygous mutant (+/dsod3^KG^) and homozygous mutant (dsod3^KG^/dsod3^KG^). Second lane: control without RNAi induction (+/dsod3R2) compared with females with ubiquitously (actGal4/dsod3R2) or ovarian-specific (OvGal4/dsod3R2) downregulated dsod3. (**G**,**H**): egg laying rates (per female per 24 h) of different genotypes with diminished dsod3 level (*n* ≥ 20 females for each genotype). (**I**,**J**): hatching rates of the different genotypes with diminished DSOD3 level (*n* = 3). Error bars in E and F mark the standard deviation (s.d.). An unpaired *t*-test provided information about significance. * = *p*-value ≤ 0.05; ** = *p*-value ≤ 0.01; *** = *p*-value ≤ 0.001; n.s. = not significant.

To analyze the in vivo function of dNox and dSod3 in ovaries, we used the hypomorph mutant line dsod3^KG06029^ (dsod3^KG^) and additionally knocked down dnox or dsod3 expression ubiquitously as well as specifically in the follicle epithel of the developing egg chambers using the Gal4/UAS-RNA interference (RNAi) system. For the RNAi experiments, two different driver lines, actGal4 (which expresses ubiquitously) and OvGal4 (which expresses specifically in follicle cells covering the developing oocyte), were used, along with two independent RNAi lines each for dsod3 (dsod3R1 and dsod3R2) and dnox (dnoxR1 and dnoxR2), respectively (see more tested ovarian-specific driver lines in Figure S4). As a control, w^1118^ (+/+) females were used. We examined female abdominal morphology, ovarian morphology, egg morphology, egg laying rate, and hatching rate of either heterozygous (+/dsod3^KG^) or homozygous (dsod3^KG^/dsod3^KG^) individuals and of females with either ubiquitous or ovarian-specific downregulated dsod3 (Figure 6).

As can be seen in Figure 6A–C, the abdomens of the heterozygous (+/dsod3^KG^) and homozygous mutants (dsod3^KG^/dsod3^KG^) are enlarged when compared with the control (+/+). The same tendency can be seen in the morphology of the dissected ovaries (Figure 6A′–C′). Both mutants contain a higher number of mature egg chamber stages than the control. The morphology of the laid eggs does not show any obvious differences from the control (Figure 6A″–C″). Similarly, females with ubiquitously or ovarian-specific downregulation of dsod3 (Figure 6E,F) show an enlarged abdomen compared with controls (Figure 6D). This can be seen even more clearly in the morphology of the ovaries (Figure 6E′,F′). Females with downregulateddsod3 contain a higher number of mature egg chamber stages in their ovaries compared with controls. The tendencies were reproduced with the second dsod3-RNAi line (see Supplementary Figure S5). Interestingly, the egg-laying rate of the dsod3^KG^ mutants was significantly different. While heterozygous (+/dsod3^KG^) females laid down nearly as many eggs per 24 h as control females, the egg-laying ability of homozygous (dsod3^KG^/dsod3^KG^) females was lowered by about 50% (Figure 6G). Furthermore, the hatching rate of the eggs laid by homozygous (dsod3^KG^/dsod3^KG^) females was significantly reduced by about 40% compared with eggs laid by heterozygous or a control female (Figure 6I).

RNAi knockdown of dsod3 diminished the egg laying rate by about 30–40%, independent of ubiquitous or ovarian-specific downregulation (Figure 6H), while the hatching rate was not markedly affected when dsod3 was RNAi downregulated (Figure 6J).

In summary, we could show that a diminished level of dsod3 expression is connected to a lowered egg-laying rate. The observation that ovarian-specific RNAi knockdown of dsod3 can impair the egg-laying process reveals an ovarian-specific function for dsod3 at the follicular epithel. 

**Figure 7:**
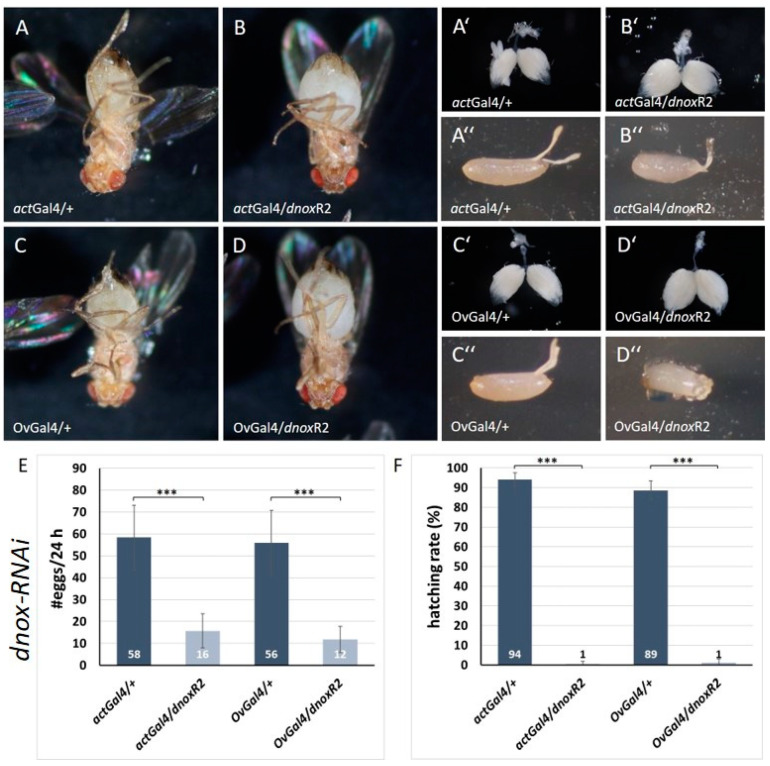
Diminished levels of dnox have an impact on ovary function, egg laying, and chorion development. (**A**–**D**): abdominal morphology is enlarged in females with ubiquitously (actGal4/dnoxR2) or ovarian-specific (OvGal4/dnoxR2) downregulated dnox compared with the controls (actGal4/+ and OvGal4/+). (**A′**–**D′**): Ovaries with ubiquitously (actGal4/dnoxR2) or ovarian-specific (OvGal4/dnoxR2) downregulated dnox are enlarged and contain more late-stage egg chambers compared with their controls (actGal4/+ and OvGal4/+). (**A″**–**D″**): Naturally laid eggs from females with ubiquitously (actGal4/dnoxR2) or ovarian-specific downregulated dnox (OvGal4/dnoxR2) are smaller and show dysmorphic chorion structures compared with the morphology control (actGal4/+ and OvGal4/+). (**E**): egg laying rates per female per 24 h of different genotypes with diminished dnox level (*n* ≥ 25 females for each genotype) (**F**): hatching rates of the different genotypes with diminished dnox levels (*n* = 3). Error bars in (**E**) and (**F**) mark the standard deviation (s.d.). An unpaired *t*-test provided information about significance. *** = *p*-value ≤ 0.001.

Using downregulation of dNox by RNA interference, we also analyzed the impact of dNox on ovarian tissue. As for dsod3, we investigated the female abdominal morphology, ovarian morphology, egg laying rate, egg morphology, and hatching rate of either females with ubiquitously downregulated dnox (actGal4/dnoxR2) or ovarian-specific downregulated dnox (OvGal4/dnoxR2) (Figure 7).

As can be seen, the abdomen of females with ubiquitously downregulated dnox (Figure 7B) and with ovarian-specific downregulated dnox (Figure 7D) is enlarged compared with the controls (Figure 7A,C, respectively). The same differences can be seen in the morphology of the ovaries. Females with downregulated dnox (Figure 7B′,D′) show enlarged ovaries compared with their controls (Figure 7A′,C′). Interestingly, contrary to the eggs laid by females with downregulated dsod3, a diminished dnox causes significant morphological differences in the eggs. Eggs laid by females with downregulated dnox (Figure 7B″,D″) have a mis- or underdeveloped chorion structure or even no chorion at all, while empty chorion particles could be found in proximity to the chorion-lacking eggs. The same results were obtained with the second dnox-RNAi line (see Figure S5).

The egg-laying rate of females with downregulated dnox is significantly different. While control females (actGal4/+ or OvGal4/+) lay about 50–60 eggs per 24 h, females with downregulated dnox lay about 10–20 eggs during the same time frame (Figure 7E). Furthermore, the hatching rate of the eggs laid by dnox-downregulated females is nearly depleted (1%) compared with eggs laid by control females that hatch at a rate of about 90% (Figure 7F).

As there is no mutant allele of the dnox gene available, we could not perform a comparative analysis like we did for dsod3. But taken together, ubiquitous and ovarian-specific downregulation of dnox in main body follicle cells, which cover the growing oocyte, seems to have a significant impact during late egg chamber processing. As dissection of the flies revealed enlarged ovaries with a high number of mature egg chambers when dnox is downregulated by RNAi, we assume that oogenesis until late-stage egg chambers is not affected by dnox knockdown, an observation that enforces earlier results [28]. The morphological inspection of the late-stage eggs in the ovary itself initially provided no evidence of defective development in the final stage of oogenesis, but the peculiar morphology of the laid eggs in the case of dnox downregulation aroused further investigation of the late-stage eggs in the ovary.

**Figure 8:**
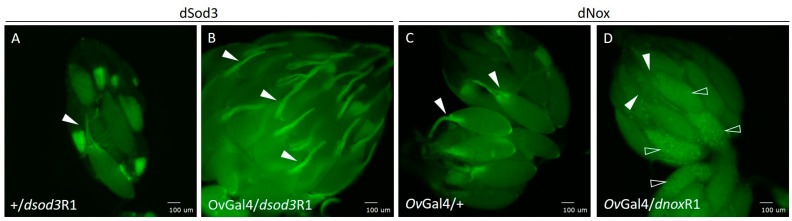
Chorion dysmorphology in dnox-downregulated ovaries. (**A**): control ovary (+/dsod3R1), (**B**): ovary with ovarian-specific downregulation of dsod3 (OvGal4/dsod3R1), and (**C**): control ovary (OvGal4/+) show fluorescence signals at chorion structures (white arrowheads). (**D**): ovary with ovarian-specific (OvGal4/dnoxR1) downregulation of dnox. All ovaries were tested for intrinsic fluorescence signals after excitation at 470 nm and with a GFP-specific emission filter set. White arrowheads point to the dorsal appendages of the chorion, and unfilled white arrowheads point to the yolk of mature egg chambers. Scale bars: 100 µm.

Interestingly, we observed a difference in the intrinsic fluorescence signal in the chorion structures of mature egg chambers in ovaries with downregulated dnox (Figure 8C,D). This difference was not detected in the chorion structures of mature egg chambers with downregulated dsod3 (Figure 8A,B).

Both control ovaries (+/dsod3R1; Figure 8,A) and ovaries with ovarian-specific downregulation of dsod3(OvGal4/dsod3R1; Figure 8B) show an intrinsic fluorescence signal at the dorsal appendages, a chorionic structure of late-stage egg chambers. Control ovaries without dnox RNAi downregulation (OvGal4/+; Figure 8C) also have an intrinsic fluorescence signal within the chorionic structures of late-stage egg chambers. In contrast, ovaries with downregulation of dnox (Figure 8D) show no intrinsic fluorescence signal of the respective chorion structures (Figure 8D, white arrowheads) and an altered yolk structure compared with control (Figure 8D, unfilled white arrowheads). The yolk of mature eggs in dnox-downregulated ovaries seems more granulated compared with the control (Figure 8C,D).

Taken together, a failure in chorion development that comes along with a unique yolk structure may be the possible cause of impaired egg laying and the disturbed chorion structure of the laid eggs. These effects were also observed using alternative ovarian-specific driver lines (see Figure S7).

### 2.3. dSod Activity within dsod3^KG^ Ovaries

As shown above, the morphology of the ovaries and egg-laying behavior are impacted by the dsod3^KG^ mutation. This insertion mutant has been described as having an effect on dsod3 expression and function, as it has shown a negative impact on the overall Cu/Zn-dSod activity in whole fly homogenates [25]. Neither immunostaining using dSod3 antibodies revealed differences in dsod3^KG^ ovaries compared with control, nor an ELISA test on ovary homogenates showed significant differences in the amount of dSod3 in the dsod3^KG^ mutant compared with wildtype control. Therefore, we concluded that the cause might not be the amount of dSod3, but the activity of the enzyme. Therefore, we assumed that the analysis of the dSod activity in the ovarian tissue would provide insight into the correlation of the amount of active enzyme with the deficiencies in fecundity. 

Previous studies estimated the total dSod activity of all three dSod isoforms or Cu/Zn-dSod activity (by inactivation of Mn-dSod) from whole adult fly samples [25].

**Figure 9:**
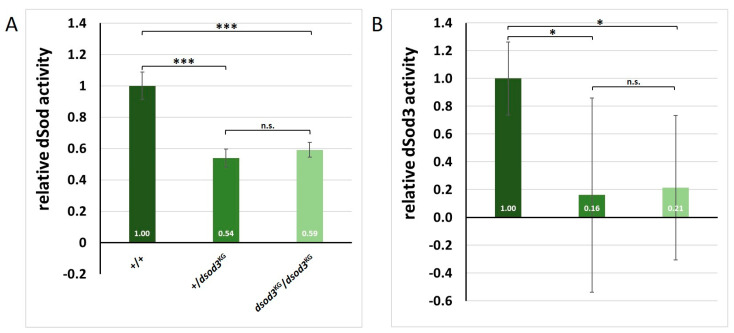
Total dSod activity compared with extracellular dSod3 activity in *Drosophila* ovaries. (**A**): Relative dSod enzyme activity within ovary homogenates of the control (+/+), the heterozygous mutant (+/dsod3^KG^), and the homozygous mutant (dsod3^KG^/dsod3^KG^); *n* = 5 for each genotype. (**B**): Relative extracellular dSod3 activity from intact ovaries of control (+/+), the heterozygous mutant (+/*dsod*3^KG^), and the homozygous mutant (*dsod*3^KG^/*dsod*3^KG^); *n* = 8 to 10 for each genotype. Values are normalized to control. Error bars in (**A**,**B**) mark the standard deviation (s.d.). An unpaired *t*-test provided information about significance. * = *p*-value ≤ 0.05; *** = *p*-value ≤ 0.001; n.s. = not significant.

Our goal was to estimate total dSod activity directly in the tissue, and we could show that the dsod3^KG^ mutation has physiological/functional consequences. This assay was realized using homogenates of ovaries (Figure 9A). By doing so, we could show a reduced overall dSod activity in the dsod3^KG^ mutant, but it was impossible to differentiate between the dSod isoforms (dSod1-3) and which of the isoforms was relatable to the measured dSod activity. 

The relative dSod activity in homogenized ovaries of the dsod3^KG^ mutant is significantly lower compared with the wildtype control (+/+) (Figure 9A). However, no significant difference in total dSod activity (all three dSod isoforms) could be detected between homozygous (dsod3^KG^/dsod3^KG^) and heterozygous (dsod3^KG^/+) mutant ovaries.

As the dSod3 isoform is predicted extracellularly, we then established a new dSod3 assay using intact ovaries (Figure 9B). By doing so, it was possible to determine if ovaries show extracellular dSod3 activity and to differentiate this activity from the intracellular dSod1 and dSod2 activity measured in homogenates. Focusing on the relative activity of extracellular dSod3, we could see a reduction of about 80% in ovaries from the heterozygous and homozygous (dsod3^KG^/dsod3^KG^) mutants compared with the control (+/+) (Figure 9B). 

### 2.4. Redox Differences in Ovaries

**Figure 10:**
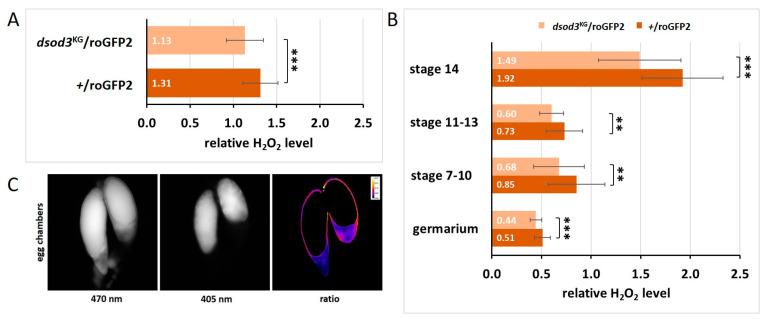
Endogenous H_2_O_2_ levels reduced in the ovaries of the dsod3^KG^ mutant. (**A**) Relative H_2_O_2_ level in whole ovaries measured by a genetically encoded roGFP2-Orp1-H_2_O_2_-sensor in a plate reader (*n* = 55 per genotype) (**B**): Relative H_2_O_2_ level in different stages of egg chamber development measured on a fluorescent microscope (*n* ≥ 30 for each genotype and egg chamber stage) (**C**): Example of egg chambers expressing the sensor molecule. Fluorescence was excited at 470 nm and at 405 nm and was always detected at 500–550 nm. The ratio of both values reveals the oxidation status of the sensor molecule (and therefore the relative H_2_O_2_ level). Error bars in (**A**,**B**) mark the standard deviation (s.d.). An unpaired *t*-test provided information about significance. ** = *p*-value ≤ 0.01; *** = *p*-value ≤ 0.001.

After having shown that dSod3 activity is significantly reduced in dsod3^KG^ mutant ovaries, we aimed to test changes in relative H_2_O_2_ levels in whole ovaries and at different stages of individual egg chambers. To obtain information about the endogenous H_2_O_2_ level, we utilized a genetically encoded redox-sensitive green fluorescent protein 2 sensor (roGFP2), specific for hydrogen peroxide, as the roGFP2 is coupled to a microbial H_2_O_2_ sensor oxidant receptor peroxidase 1 (Orp1) [36,37]. We crossed homozygous biosensor-transgenic flies (BL67670; with roGFP2-Orp1-H_2_O_2_-sensor expressed ubiquitously through the tubulin promoter) with homozygous dsod3^KG^ mutant flies or with wildtype as a control. Ovaries of the resulting F1 females (heterozygous for the sensor and for the dsod3^KG^ mutation) were dissected and either measured as whole ovaries in a plate reader-based assay (Figure 10A) or single egg chambers were dissected and imaged using fluorescence microscopy (Figure 10B,C).

Ovaries of both genotypes were tested for sensor functionality by determining their responsiveness to exogenous reduction and oxidation by exposure of freshly prepared ovaries to the exogenous reductant (dithiothreitol, DTT) for maximum reduction or the exogenous oxidant (diamide, DA) for maximum oxidation of the tissue. Both genotypes showed a similar dynamic range (DR; ratio of Ox_max_/Ox_red_) of about 1.65. Therefore, the dsod3^KG^ mutation seems to have no impact on the functionality of the sensor itself. The reduced gene dose in the heterozygous dsod3^KG^ mutant ovaries (dsod3^KG^/roGFP2) led to a 14% decrease in H_2_O_2_ levels compared with control ovaries (+/roGFP2) (Figure 10A). 

A similar significant difference of about 14% was also given in each stage of the analyzed individual egg chambers (Figure 10B). Interestingly, late-stage egg chambers (stage 14) showed a two- to three-fold higher H_2_O_2_ level compared with earlier stages. In single egg chambers, DR was about 2.14 (+/roGFP2) and 2.69 (dsod3^KG^/roGFP2). The area that was considered for redox measurement within a single egg chamber always excluded the yolk, as can be seen in the ratio image (Figure 10C). This was necessary, as with the given excitation wavelength, the intrinsic fluorescence of the yolk would have led to incorrect results. Therefore, we focused on H_2_O_2_ levels within the follicular epithel around the growing oocyte and the nutrient cells. Taken together, the H_2_O_2_ levels intrinsically increase within late-stage egg chambers, and a reduced dsod3 gene dose in dsod3^KG^ mutants relates to reduced H_2_O_2_ levels within the above-mentioned areas.

## 3. Discussion

The development of human ovarian dysfunctions like endometriosis, polycystic ovary syndrome (PCOS), oocyte stress/aging and unexplained infertility is often directly associated with an imbalance between oxidants and antioxidants [38,39]. However, due to ethical and practical obstacles, it is difficult to learn adequately about both the physiological role of ROS and the pathological development processes associated with oxidative stress during human reproduction. Over the last decade, *Drosophila* has been used successfully as a model organism in the context of ovarian (mis)functions [18,40,41,42]. Genes involved in *Drosophila* ovarian function have remained highly conserved throughout evolution [43], and even cellular organization and oocyte development show similarities [18].

In this study, the impact of the ROS-producing NADPH oxidases (NOX/dNox) and the antioxidative extracellular superoxide dismutases (EC-SOD/dSod3) on ovarian function was analyzed. We further examined the localization of the two enzymes using a newly generated dNox antibody and a dSod3 antibody that was originally used in human tissue. However, although the experiments conducted indicate the specificity of the dNox antibody, further confirmation is still needed.

The *Drosophila* NADPH oxidase (dNox) represents the orthologue of human NOX5. The function of NOX proteins in the human female reproductive organs is still unclear, but NOX4 and NOX5 proteins were shown to be localized in the human ovary [44,45,46] and were described in human granulosa cells by RT-PCR [47]. 

Previous studies narrowed down the localization of *Drosophila* dNox indirectly to the plasma membrane of follicle cells in mature egg chambers (stage 14) [28], and single-cell RNA sequencing identified dNox expression in follicle cells [48].

Our results agree with previous RNA sequencing data and expand the preliminary localization of previous studies to a more widespread localization of dNox in the follicular epithelium of developing mid- to late-egg chambers and the oviduct. 

We show that RNAi-downregulation of *dnox* expression in this follicular epithelium leads to a diminished number of laid eggs and rough chorionic defects, finally resulting in a non-viable progeny. To evaluate the effect of RNAi on the expression of the dnox gene, we used RT-qPCR analysis on RNA from actGal4 > dnoxRNAi ovary extracts. Unexpectedly, the application of actGal4 > dnoxRNAi resulted in an increase in the dnox expression rate in ovarian tissue. As this does not seem to lead to an increase in the total amount of dNox protein in ovarian tissue, as shown in the western blot in Figure S1, a compensation mechanism may be involved that responds to the RNAi of *dnox* expression, but we were unable to elucidate these further.

Reduced egg laying and ovulation rates because of inducing *dnox* RNAi have been described before [28,34], but none of the previous studies reported chorionic defects or a diminished hatching rate. Instead, Ritsick et al. describe that eggs laid by tubulinPGal4 > UAS*dnox*-RNAi females have normal hatching rates and are fully viable [34]. In order to rule out that the chorionic defects and the radically minimized survival rate in our experiments are a coincidental finding or are specifically due to the one ovarian driver strain used, we tested additional ovarian-specific driver lines (see Table S1). Except for *slbo*Gal4 (BL6458), all ovarian-specific driver lines and the ubiquitous driver line *act*Gal4 (BL3954) led to an extremely reduced laying rate and a disabled hatching with non-viable progeny. These results enforce that the function of dNox in follicular cells surrounding the maturing oocyte is essential. 

In previous studies, seemingly intact eggs from the ovaries of *dnox*-RNAi females could be dissected and subsequently subjected to a rupture test in vitro [28], without any further abnormalities. In contrast, we show differences yet in the developing chorion structure and in the yolk of corresponding late-stage egg chambers. 

We also tested 47A04Gal4 (BL50286), the driver line that was used in previous studies to knockdown *dnox* in follicle cells in only mature late-stage 14 egg chambers [28]. This knockdown led to reduced in vitro follicle rupture in dissected eggs. In contrast, our experiments to knockdown *dnox* with this driver line showed neither morphologic changes of the ovaries (see Figure S6), only slightly reduced egg laying, nor morphologically impacted eggs. This might be due to the experimental conditions, as Li et al. performed all RNAi knockdown experiments under amplifying conditions at 29 °C and with co-expression of additional Dicer2 (*dcr2*) protein [28]. All our *dnox* RNAi knockdown experiments were performed at 25 °C. Additionally, all ovary-specific driver lines we used targeted follicle cells in a broader range, from early to mid-stage egg chambers. Therefore, we believe that we revealed dNox functions at earlier stages because stronger effects occurred this way. This is consistent with our localization results for dNox in the mid-stage egg chamber stages as well.

Considering dNox produces superoxide anions (O_2_^●−^) in the extracellular space, the loss of these reactive oxygen species (in the case of dNox downregulation) may cause the above-mentioned morphological consequences on the entire chorion structure. Otherwise, the superoxide anions may usually be involved in further reactions, leading to molecules like peroxynitrite (ONOO^−^) or hydrogen peroxide (H_2_O_2_) [49].

The appearance of hydrogen peroxide in *Drosophila* egg chambers has been described at the end of the eggshell assembly, where it is supposed to be the natural trigger of the peroxidase (Pxd)-catalyzed chorion protein crosslinking [50,51,52]. Through this hardening process, the chorion becomes insoluble and is able to protect the laid egg from desiccation. The source of the needed hydrogen peroxide is not fully understood, as it has already been proven chemically, but the biological source has never been precisely named [53,54]. We suppose that the missing source of hydrogen peroxide might be the dNox-produced superoxide anions, which might be dismutated by extracellular dSod3 to hydrogen peroxide. Therefore, the lack of superoxide anions due to *dnox* knockdown could lead to morphological changes in the chorion, as we observed. 

The localization of the Pxd is described mainly in the respiratory filaments, in the micropyle area, and with a lower content in the chorion structure of stage 14 follicles [51], matching the localization of dNox and dSod3 we found in this work. Additionally, in previous studies, the use of a peroxidase inhibitor suppressed egg laying, which resulted in chorions that were cracked during egg delivery [55], similar to the observations in our work.

Furthermore, since the reduced H_2_O_2_ level we proved within the egg chambers of the *dsod*3^KG^ mutant and the RNAi-knockdown of *dsod3* did not result in chorionic defects, as they were observed when *dnox* is knocked down, we assume that dNox works upstream of the extracellular dSod3. A loss of dNox has a greater physiological impact than the loss of dSod3 because dNox produces the substrate for the extracellular dSod3. At first, dNox produces superoxidanions in the extracellular space that get dismutated to H_2_O_2_ by extracellular dSod3. Second, the so-generated H_2_O_2_ molecules may in part enter the follicle cells or get used extracellularly for chorion hardening processes.

Even with extremely reduced dSod3, superoxide anions would still be produced by dNox, and they could spontaneously dismutate to H_2_O_2_, or they could be sufficiently dismutated by the remaining dSod3 function. Whereas a depletion of *dnox*, the primary source of superoxide anions, may have a greater effect on lowering H_2_O_2_ levels and therefore lead to more severe consequences. This would be in accordance with our observations.

However, we have not yet been able to measure the H_2_O_2_ level in *dnox* knockdown ovaries, as co-expression of both constructs (roGFP2-sensor and UAS-*dnox*RNAi) led to lethality. For future experiments, it might be interesting to recombine the roGFP2-sensor with the *dsod*3^KG^ mutation. An appropriately established strain would open up the possibility of determining the H_2_O_2_ levels in the homozygous *dsod*3^KG^ mutation and combining the *dsod*3^KG^ mutation with additional mutations or constructs, e.g., concerning dNox.

Extracellular hydrogen peroxide production was also described as a critical step for in vitro follicle rupture [28]. Within the follicle rupture process, extracellular dSod3 was suggested to convert dNox-produced superoxide to hydrogen peroxide, but it remained unclear whether hydrogen peroxide acts extracellularly or diffuses through the cell membrane to reach targets for follicle rupture [28]. Since the roGFP2 sensor used in our study is expressed cytoplasmically, the observed reduction in hydrogen peroxide level (in *dsod3*^KG^ mutants) should be attributed to the cytoplasm, suggesting that the hydrogen peroxide has at least partially penetrated the corresponding cells. However, this does not rule out that the hydrogen peroxide produced extracellularly does not have extracellular functions.

The *Drosophila* superoxide dismutase 3 (dSod3) represents the orthologue to the human SOD3, or EC-SOD. High expression of human Cu/Zn-SOD was detected in granulosa cells and theca interna cells of antral and dominant follicles, as well as in luteinized granulosa and theca cells of functioning corpora lutea [7,15,56], while extracellular SOD3/EC-SOD was shown in bovine cumulus cells and in cultured cumulus cell/oocyte complexes (COC), where it is the only SOD isoform that can be detected in the zona pellucida [57]. EC-SOD was also shown to be a critical regulator of the COC’s extracellular matrix (ECM) [12,58]. Due to the lack of useful antibodies, little is known about the localization of dSod3 in the reproductive tract of *Drosophila*: the *dsod3* expression is elevated in ovaries [26], it has been shown that dSod3 is enriched in late-stage egg chambers [27], and overexpression of dSod3-HA in follicle cells led to the assumption of an extracellular localization of dSod3 in the flies’ ovaries [28].

Our results show a localization of dSod3 in the follicular epithelium of developing egg chambers, in the epithelial muscle-sheath of the ovarioles, and in the muscle layer of the oviduct. Accordingly, there is a similar localization of both enzymes in the mammalian granulosa cells/in the zona pellucida, which in *Drosophila* corresponds to the follicular cells/the space between the follicular cells and the egg cell. The localization of dSod3 at muscle sheaths might represent the predicted membrane-bound isoform [25] and may correspond to the smooth muscle cells associated with SOD3 [59].

Previous studies described a dNox depletion-associated inhibition of muscle contractions in the ovaries [34], which led us to suggest that the spatial cooperation of dNox and dSod3 may play a role within the muscle sheaths. Our localization experiments revealed that both enzymes are present in the oviduct. As the ovulation rate was markedly lower in *dnox* RNAi females, previous studies concluded a defective movement of the egg from the ovary into oviducts and showed markedly attenuated muscle contractions in *dnox*-depleted ovaries [34]. The muscles surrounding the gut and gonads are functionally analogous to vertebrate smooth muscle [60]. There are three muscle types in the ovary: the peritoneal sheath surrounding each of the two intact ovaries and the epithelial sheath, a thin circular muscle that surrounds each of the approximately 16 ovarioles, both of which derive from mesodermal cells. Lateral oviducts, which fuse into the common oviduct through which mature eggs pass to enter the uterus, are surrounded by a circular layer of somatic muscles [60].

The precise mechanisms inducing muscle contraction and relaxation in the *Drosophila* ovaries are not known, but previous studies show that both processes are regulated by octopamine via two receptors, OAMB and Octß2R, that are expressed in the epithelia underneath the muscle layer of the entire oviduct [61] and the follicle cells surrounding the devloping egg [62]. Octopamine/OAMB induces a rise in intracellular Ca^2+^ in these cells [61,62,63]. Octopamine/OAMB-Ca^2+^ is then able to activate dNox to produce superoxide anions in the extracellular space [28]. The role of dNox-derived superoxide anions is still not clear. Getting dismutated to hydrogen peroxide may be important for the rupturation process, as stated in [28]. However, superoxide anions might also have an impact on contraction or relaxation processes in the oviduct muscles. A role for Ca^2+^-sensitive NOX5 in redox regulation of smooth muscle contraction has been shown [64].

Assuming the smooth-like muscles of the oviduct are sensitive to nitric oxide (NO^●^) signaling, extracellular dSod3 activity could regulate the contraction or dilation process by modulating the dNox-produced superoxide ion levels, as they are able to switch off the nitric oxide response [49]. NO-signaling is known in mammalian ovarian physiology, as it has been demonstrated that the multiple isoenzymes of nitric oxide synthase (NOS) are expressed and localized in the ovary of different species [65,66]. A role for NO was ascribed to the processes of steroidogenesis, folliculogenesis, oocyte meiotic maturation, and ovulation [65,67,68]. SODs in mammals are known to modulate the bioavailability of nitric oxide and peroxynitrite (ONOO-) rate at vascular smooth muscles or, hence, in the extracellular space [49,69].

Therefore, superoxide ions, which normally switch off the nitric oxide response, could be overrepresented when dsod3 is downregulated in oviducts, leading to an impaired dilatation of the oviduct musculature. That might impact the contraction/dilation of the lateral or common oviduct and, therefore, block the exit for the mature follicles. This would be consistent with the significantly reduced egg-laying rate we observed with the homozygous dsod3KG mutant and the dsod3 RNAi experiments. However, a role for nitric oxide in the Drosophila reproductive tract has not been described yet. In future experiments, it would be interesting to investigate the NO synthase function in Drosophila ovaries.

Regarding the hatching phenotype, in which the effect of the homozygous *dsod*3^KG^ mutation and the influence of *dsod*3RNAi differ significantly, it remains to be finally clarified whether the specificity is due to the *dsod*3 mutation or if it might come from an unknown genetic background mutation. Using another *dsod*3 mutant allele, or *dsod*3^KG^, in combination with a deficiency encompassing the *dsod*3 locus might provide clarity but was not the main focus of the study.

## 4. Conclusions

In this study, we show that in the female reproductive tissue, the localization of dNox and dSod3 largely corresponds to the localization of the mammalian orthologous NOX5 and EC-SOD. This forms a good basis for exploring the function of their mammalian counterparts using the model organism *Drosophila*. Our results indicate a collaboration between the two enzymes dNox and dSod3, not only during the egg hardening process but also during the passage of mature eggs through the oviduct.

## 5. Materials and Methods

### 5.1. Antibodies

The dNox antibody was generated against a synthesized peptide according to the amino acid sequence of the C-terminal end (aa 1325 to aa 1340). Synthetization of the peptide and immunization process was done at GeneCust Service (Boynes, France). The antigen region can be found in all annotated isoforms of dNox (dNox-PB, -PC, -PD, and -PE), making the antibody detect all of them. Cross-reaction of the antibody with the second (dual-specific) NADPH oxidase (dDuox) was avoided by checking amino acid sequences with alignments and by blast. 

### 5.2. Immunoblotting and Immunostaining

For western blotting analyses, ovaries were freshly prepared in ice-cold phosphate buffered saline (1X PBS: 137 mM NaCl, 2.7 mM KCl, 2 mM KH_2_PO_4_, 1.8 mM Na_2_HPO_4_·2H_2_O), transferred to homogenization buffer (1X PBS with 1% sodiumdodecylsulfate (SDS), and manually homogenized with a pistil on ice and an additional 3X10 sec sonification step (5 ovaries per 200 µL). Loading buffer was added to the homogenates, and the probes were boiled at 95 °C. Afterwards, probes were separated on SDS-polyacrylamide gel electrophoresis (PAGE) and blotted on nitrocellulose membrane. Blocking was done with 5% skimmed milk powder. For immunoblotting, the dNox antibody (1:250; this work), dSod3 antibody (1:250; Invitrogen, Waltham, MA, USA; #PA5-102904), and α-Tubulin antibody (1:4000; Sigma-Aldrich, Taufkirchen, Germany; #6144) were used and detected with peroxidase-conjugated secondary anti-rabbit (Sigma; #A0545) or anti-mouse (Sigma; #A9044) antibody (1:20.000) and ECL western blotting substrate (Thermo Scientific^TM^ Pierce^TM^; Waltham, MA, USA; #32106). Incubation with primary antibodies was performed over night at 8 °C and with secondary antibodies for 2 h at room temperature (RT). Images were taken using Invitrogen^TM^ iBright^TM^ FL1500 imaging system (Waltham, MA, USA) and iBright^TM^ FL1500 imaging system software.

For immunostaining, ovaries were freshly prepared in ice-cold 1X PBS, fixed with 4% paraformaldehyde (PFA) for 1h at RT, rinsed in high salted 1X PBS_hs_ (500 mM NaCl, 2.7 mM KCl, 1.8 mM KH_2_PO_4_, 10.1 mM Na_2_HPO_4_·2H_2_O), washed repeatedly in 1X PBS_hs_ with 0.5% Tween 20 (1X PBST_hs_), and blocked with 1XPBST_hs_ with 5% fetal calf serum (FCS) for 30 min at RT. For immunostaining, the dNox antibody (1:500; this work) and the dSod3 antibody (1:500; Invitrogen #PA5-102904) were used and detected with fluorophore (Cy^TM^3 or CY^TM^5) conjugated secondary anti-rabbit antibodies (1:500; Jackson Immunoresearch; Baltimore, PA, USA; #111-165-003 and #111-175-003). Incubation with primary antibodies was performed over night at 8 °C and with secondary antibodies for 2 h at RT. Ovaries were mounted in Vectashield antifade mounting medium (Vectorlabs; Newark, CA, USA). In each staining, ovaries were included as a technical control (blank) that were treated exactly the same but without the primary antibody. Images were taken with an Axiovert 200 M fluorescence microscope (Zeiss; Oberkochen, Germany) and Slidebook 6 software. TIFF images were processed with the software ImageJ (NIH, Bethesda, MD, USA).

### 5.3. Fly Strains

Strains obtained from the Bloomington *Drosophila* Stock Centre (BDSC; given with BL number) or Vienna *Drosophila* Research Centre (VDRC; given with VDRC ID number) are: *dsod3*^KG06029^ (BL14138); UAS-*dsod3*R1 (VDRC8760); UAS-*dsod3*R2 (VDRC37793); UAS-*dnox*R1 (BL32433); UAS-*dnox*R2 (BL32902); *act*Gal4 (BL3954); OvGal4 (BL3750; also known as “c355Gal4”); *slbo*Gal4 (BL6458); CY2Gal4 (BL93734); GR1Gal4 (BL36287); 47A04Gal4 (BL50286; enhancer trap line from janelia campus); w^1118^ (BL5905); YW (BL6599); tub-cyto-roGFP2-Orp1 (BL67670; [37]); CRIMIC-*dnox*Gal4 (BL78988; [70]); UAS-mCD8-cherryRFP (BL27392).

### 5.4. Egg Laying/Egg Hatching Analysis

To get females with the required genotype, crossings were done by putting equal amounts of male and female flies of the same age together and keeping them in vials under standard conditions (25 °C, 65% humidity, 12/12 dark light cycle). Resulting virgin F1 females were separated and backcrossed with equal amounts of males from the control strain (w^1118^or YW). Backcrosses (*n* = 3 for each genotype) were kept in egg-laying cups for up to 5 days at standard conditions, holding apple juice agar plates with equal amounts of yeast paste that were changed twice every 24 h. Since the flies need an acclimatization period until a constant egg-laying rate is reached, the first 24 h after the crossing were not included in the evaluations. For the evaluation, at least double determinations were carried out for each backcross by counting days 2 (24–48 h after crossing) and 3 (48–72 h after crossing). Laid eggs on each plate were counted to estimate the egg-laying rate per female in 24 h of each genotype, then the plates were kept another 24–72 h at standard conditions. Hatched larvae on each plate were counted to estimate the hatching rate of each analyzed genotype. Female flies from the egg-laying cups were documented for abdominal morphology, and subsequently prepared ovaries were documented for ovarian morphology. Eggs were photographed on apple juice agar plates to document egg morphology. All images were taken with the help of a binocular and a Canon EOS-RP camera under consistent conditions.

### 5.5. dSod Activity Assay/dSod3 Activity Assay

Total dSod activity in ovarian tissue was examined using a modified Sod activity assay based on [71]. For each sample, five freshly dissected ovaries were manually homogenized and dissolved in 350 µL phosphate buffered saline (PBS; 137 mM NaCl, 2.7 mM KCl, 2 mM KH_2_PO_4_, and 1.8 mM Na_2_HPO_4_·2H_2_O), followed by a serial dilution. Each well of a 96-well plate was preloaded with 110 µL of reaction solution (PBS containing 100 µM hypoxanthine and 300 µM water-soluble tetrazolium salt (WST-1)). In each preloaded well, 70 µL of a sample dilution were added before starting the enzymatic reaction by adding 20 µL of xanthine oxidase (0.0125 U/mL) per well. 

As superoxide anions are produced when hypoxanthine is degraded by the xanthine oxidase, WST-1 is transformed into WST-1-formazan, which is a yellow dye that can be detected by optical density measurement in a plate reader. The higher the total dSod activity in the samples, the lower the production of WST-1-formazan. Measurements were performed in 96-well plates with the Spark multimode microplate reader (Tecan Group; Männedorf, Switzerland) at a wavelength of 450 nm using 620 nm as reference and at 25 °C. Since ovaries of the three genotypes differ in size, the dSod activity per amount of protein was determined. For this, BCA protein quantification (ThermoFisher Scientific; Pierce^TM^ BCA Protein Assay Kit, #23225) in duplicates of each sample dilution was done separately.

Furthermore, we established a modified dSod3 activity assay to be able to distinguish between intra- and extracellular dSod activity. Each assay was carried out, measuring the activity of the extracellular dSod3 of a single intact ovary per well. Each well was preloaded with 188 µL reaction solution (*Drosophila* saline (DS; 130 mM NaCl, 5 mM KCl, 2 mM MgCl_2_, 2 mM CaCl_2_, 5 mM HEPES, 36 mM sucrose, 5 mM glucose; pH 7.0 at 25 °C) containing 100 µM hypoxanthine and 300 µM water-soluble tetrazolium salt (WST-1)). To start the reaction, 12 µL of xanthine oxidase (0.05 U/mL) was added per well, and measurement was immediately started. Readings were taken every minute for 5 min without ovaries to get the basal activity of the added xanthine oxidase. After 5 min, freshly in *Drosophila* saline, dissected intact ovaries were added (one per well). The higher the extracellular dSod3 activity of an ovary, the greater the inhibition of WST-1-formazan production. As a control, wells without added ovary were measured (getting the dynamic/curve of an unhindered WST-1-formazan production). Measurements were continued immediately (per minute) for 5 more minutes. Subsequently, ovaries were removed from the wells, and measurements were continued (per minute) for 5 more minutes. The evolution of the optical density was established graphically, and the relative extracellular enzyme activity of dSod3 could be determined.

### 5.6. H_2_O_2_ Sensor Measurements

H_2_O_2_-levels in whole ovaries and in different stages of single egg chambers were observed by using transgenic flies ubiquitously expressing a genetically encoded roGFP2-Orp1-H_2_O_2_-sensor that allows measuring H_2_O_2_ (BL67670; [37]). 

For the measurement of whole ovaries, a 96-well plate (dark, conical bottom) was prepared by adding 50 µL of PBS per well. Freshly in phosphate-buffered saline (PBS: 137 mM NaCl, 2.7 mM KCl, 2 mM KH_2_PO_4_, and 1.8 mM Na_2_HPO_4_·2H_2_O), dissected ovaries were placed at the bottom into the center of the wells (one per well). Measurement was started immediately. Fluorescence was excited sequentially at 405 and 490 nm, and emission was detected at 500–560 nm. Calibration was done with the same ovaries, either fully reduced by adding dithiothreitol (DTT) to a final concentration of 2 mM or fully oxidized by adding diamide (DA) to a final concentration of 0.5 mM. Measurement was restarted immediately. Maximum reduction was reached after 5 min, and maximum oxidation was reached after 10 min. Ratio values were calculated by dividing the 405 nm value by the 490 nm value. Dynamic range (DR) was calculated by the division: ratio of maximum oxidation/ratio of maximum reduction.

For the measurement of single egg chambers, a sealing ring (ø 10/6 mm) fixed on a coverslip was prefilled with 50 µL of 1X PBS. Ovaries were dissected in 1X PBS, and staged egg chambers were transferred into the sealing ring and imaged immediately. Calibration was done with the same egg chambers, now fully reduced by adding dithiothreitol (DTT) to a final concentration of 2 mM or fully oxidized by adding diamide (DA) to a final concentration of 0.5 mM. Measurement was restarted immediately. Maximum reduction was reached after 5 min, and maximum oxidation was reached after 10 min. Imaging was done using an inverse epifluorescence microscope (Axiovert 200M, Zeiss; Oberkochen, Germany) with CoolLED pE-4000 light source, ORCA-fusion camera (Hamamatsu, Japan), and Slidebook6 imaging software (3i, Intelligent Imaging Innovations, Göttingen, Germany). Fluorescence was excited sequentially at 405 and 470 nm and detected at 500–550 nm (HQ 525/50 nm bandpass). Images were saved and exported as 16-bit tiff files and further processed in ImageJ. Paired 405 nm and 470 nm images were put into a stack. Yolk area was subtracted in both images using the 405 nm image with a matching threshold, creating a region of interest (ROI) that could be deleted in both images. Afterwards, the intensities of the 490 nm image were thresholded, and values below this threshold were set to “not a number” (NaN). Values of each channel were obtained using “multi measure” and copied to Microsoft Excel. Values were calculated as ratio (405/490 nm). The ratio image was created by subtracting background with “rolling ball” set to 150 pixels, dividing the 405 nm image by the 490 nm image pixel by pixel, and false coloring with the lookup table “fire.” All images were taken with the same microscope settings.

### 5.7. Statistical Analysis

Data representation and statistical analysis were performed using Microsoft Excel (Microsoft Corporation, Redmond, WA, USA). Unpaired student’s *t*-test was used, all data are mean values from three or more independent experiments with equal sample sizes (see figure legends for *n*), +− standard deviation (s.d.). Statistical significance was set at *p* ≤ 0.05. Asterisks indicate * *p* ≤ 0.05, ** *p* ≤ 0.01, *** *p* ≤ 0.001, n.s. = not significant.

## Supplementary Materials

The following supporting information can be downloaded at: https://www.mdpi.com/article/10.3390/ijms25116138/s1.

## References

[1] D’Autréaux B., Toledano M.B. (2007). ROS as Signalling Molecules: Mechanisms That Generate Specificity in ROS Homeostasis. Nat. Rev. Mol. Cell Biol..

[2] Finkel T. (2011). Signal Transduction by Reactive Oxygen Species. J. Cell Biol..

[3] Sies H., Belousov V.V., Chandel N.S., Davies M.J., Jones D.P., Mann G.E., Murphy M.P., Yamamoto M., Winterbourn C. (2022). Defining Roles of Specific Reactive Oxygen Species (ROS) in Cell Biology and Physiology. Nat. Rev. Mol. Cell Biol..

[4] Sies H., Jones D.P. (2020). Reactive Oxygen Species (ROS) as Pleiotropic Physiological Signalling Agents. Nat. Rev. Mol. Cell Biol..

[5] Sugino N. (2005). Reactive Oxygen Species in Ovarian Physiology.

[6] Shkolnik K., Tadmor A., Ben-Dor S., Nevo N., Galiani D., Dekel N. (2011). Reactive Oxygen Species Are Indispensable in Ovulation. Proc. Natl. Acad. Sci. USA.

[7] Wang S., He G., Chen M., Zuo T., Xu W., Liu X. (2017). The Role of Antioxidant Enzymes in the Ovaries. Oxid. Med. Cell Longev..

[8] Liang J., Gao Y., Feng Z., Zhang B., Na Z., Li D. (2023). Reactive Oxygen Species and Ovarian Diseases: Antioxidant Strategies. Redox Biol..

[9] Rizzo A., Roscino M.T., Binetti F., Sciorsci R.L. (2012). Roles of Reactive Oxygen Species in Female Reproduction. Reprod. Domest. Anim..

[10] Murphy M.P., Bayir H., Belousov V., Chang C.J., Davies K.J.A., Davies M.J., Dick T.P., Finkel T., Forman H.J., Janssen-Heininger Y. (2022). Guidelines for Measuring Reactive Oxygen Species and Oxidative Damage in Cells and in Vivo. Nat. Metab..

[11] Zelko I.N., Mariani T.J., Folz R.J. (2002). Superoxide Dismutase Multigene Family: A Comparison of the CuZn-SOD (SOD1), Mn-SOD (SOD2), and EC-SOD (SOD3) Gene Structures, Evolution, and Expression. Free. Radic. Biol. Med..

[12] Fukai T., Ushio-Fukai M. (2011). Superoxide Dismutases: Role in Redox Signaling, Vascular Function, and Diseases. Antioxid. Redox Signal.

[13] Marklund S.L. (1982). Human Copper-Containing Superoxide Dismutase of High Molecular Weight. Proc. Natl. Acad. Sci. USA.

[14] Marklund S.L., Holme E., Hellner L. (1982). Superoxide Dismutase in Extracellular Fluids. Clin. Chim. Acta.

[15] Matos L., Stevenson D., Gomes F., Silva-carvalho J.L., Almeida H. (2009). Superoxide Dismutase Expression in Human Cumulus Oophorus Cells. Mol. Hum. Reprod..

[16] Talat A., Satyanarayana P., Anand P. (2022). Association of Superoxide Dismutase Level in Women with Polycystic Ovary Syndrome. J. Obstet. Gynecol. India.

[17] Wang Y., Branicky R., Noë A., Hekimi S. (2018). Superoxide Dismutases: Dual Roles in Controlling ROS Damage and Regulating ROS Signaling. J. Cell Biol..

[18] Armstrong A.R. (2020). Reproduction Review *Drosophila melanogaster* as a Model for Nutrient Regulation of Ovarian Function. Reproduction.

[19] Landis G.N., Tower J. (2005). Superoxide Dismutase Evolution and Life Span Regulation. Mech. Ageing Dev..

[20] Hewitt O.H., Degnan S.M. (2022). Distribution and Diversity of ROS-Generating Enzymes across the Animal Kingdom, with a Focus on Sponges (Porifera). BMC Biol..

[21] Phillips J.P., Campbellt S.D., Michaud D., Charbonneaut M., Hilliker A.J. (1989). Null Mutation of Copper/Zinc Superoxide Dismutase in *Drosophila* Confers Hypersensitivity to Paraquat and Reduced Longevity. Proc. Natl. Acad. Sci. USA.

[22] Duttaroy A., Parkes T., Emtage P., Kirby K., Boulianne G.L., Wang X., Hilliker A.J., Phillips I.P. (1997). The Manganese Superoxide Dismutase Gene of Drosophila: Structure, Expression, and Evidence for Regulation by MAP Kinase.

[23] Kirby K., Hu J., Hilliker A.J., Phillips J.P. (2002). RNA Interference-Mediated Silencing of Sod2 in *Drosophila* Leads to Early Adult-Onset Mortality and Elevated Endogenous Oxidative Stress. Proc. Natl. Acad. Sci. USA.

[24] Jung I., Kim T.Y., Kim-Ha J. (2011). Identification of *Drosophila* SOD3 and Its Protective Role against Phototoxic Damage to Cells. FEBS Lett..

[25] Blackney M.J., Cox R., Shepherd D., Parker J.D. (2014). Cloning and Expression Analysis of *Drosophila* Extracellular Cu Zn Superoxide Dismutase. Biosci. Rep..

[26] Chintapalli V.R., Wang J., Dow J.A.T. (2007). Using FlyAtlas to Identify Better *Drosophila melanogaster* Models of Human Disease. Nat. Genet..

[27] Eichhorn S.W., Subtelny A.O., Kronja I., Kwasnieski J.C., Orr-Weaver T.L., Bartel D.P. (2016). MRNA Poly(A)-Tail Changes Specified by Deadenylation Broadly Reshape Translation in Drosophila Oocytes and Early Embryos. 2016. Elife.

[28] Li W., Young J.F., Sun J. (2018). NADPH Oxidase-Generated Reactive Oxygen Species in Mature Follicles Are Essential for *Drosophila* Ovulation. Proc. Natl. Acad. Sci. USA.

[29] Lambeth J.D. (2004). NOX Enzymes and the Biology of Reactive Oxygen. Nat. Rev. Immunol..

[30] Bedard K., Krause K.-H. (2007). The NOX Family of ROS-Generating NADPH Oxidases: Physiology and Pathophysiology. Physiol. Rev..

[31] Brown D.I., Griendling K.K. (2009). Nox Proteins in Signal Transduction. Free Radic. Biol. Med..

[32] Bedard K., Jaquet V., Krause K.H. (2012). NOX5: From Basic Biology to Signaling and Disease. Free Radic. Biol. Med..

[33] Touyz R.M., Anagnostopoulou A., Rios F., Montezano A.C., Camargo L.L. (2019). NOX5: Molecular Biology and Pathophysiology. Exp. Physiol..

[34] Ritsick D., Edens W., Finnerty V., Lambeth D. (2007). Nox Regulation of Smooth Muscle Contraction. Free Radic. Biol. Med..

[35] Diebold B.A., Wilder S.G., De Deken X., Meitzler J.L., Doroshow J.H., McCoy J.W., Zhu Y., Lambeth J.D. (2019). Guidelines for the Detection of NADPH Oxidases by Immunoblot and RT-QPCR. Methods in Molecular Biology.

[36] Gutscher M., Sobotta M.C., Wabnitz G.H., Ballikaya S., Meyer A.J., Samstag Y., Dick T.P. (2009). Proximity-Based Protein Thiol Oxidation by H2O2-Scavenging Peroxidases. J. Biol. Chem..

[37] Albrecht S.C., Barata A.G., Großhans J., Teleman A.A., Dick T.P. (2011). In Vivo Mapping of Hydrogen Peroxide and Oxidized Glutathione Reveals Chemical and Regional Specificity of Redox Homeostasis. Cell Metab..

[38] Agarwal A., Gupta S., Sharma R.K. (2005). Role of Oxidative Stress in Female Reproduction. Reprod. Biol. Endocrinol..

[39] Agarwal A., Gupta S., Sekhon L., Shah R. (2008). Redox Considerations in Female Reproductive Function and Assisted Reproduction: From Molecular Mechanisms to Health Implications. Antioxid. Redox Signal.

[40] Liu H., Li J., Chang X., He F., Ma J. (2022). Modeling Obesity-Associated Ovarian Dysfunction in *Drosophila*. Nutrients.

[41] Brookheart R.T., Duncan J.G. (2016). *Drosophila melanogaster*: An Emerging Model of Transgenerational Effects of Maternal Obesity. Mol. Cell Endocrinol..

[42] Bestetti I., Barbieri C., Sironi A., Specchia V., Yatsenko S.A., de Donno M.D., Caslini C., Gentilini D., Crippa M., Larizza L. (2021). Targeted Whole Exome Sequencing and *Drosophila* Modelling to Unveil the Molecular Basis of Primary Ovarian Insufficiency. Human. Reprod..

[43] Elis S., Desmarchais A., Cardona E., Fouchecourt S., Dalbies-Tran R., Nguyen T., Thermes V., Maillard V., Papillier P., Uzbekova S. (2018). Genes Involved in Drosophilamelanogaster Ovarian Function Are Highly Conserved throughout Evolution. Genome Biol. Evol..

[44] Buck T., Hack C.T., Berg D., Berg U., Kunz L., Mayerhofer A. (2019). The NADPH Oxidase 4 Is a Major Source of Hydrogen Peroxide in Human Granulosa-Lutein and Granulosa Tumor Cells. Sci. Rep..

[45] Buck T. (2018). Role of ROS and ROS Generating Enzymes in the Human Ovary.

[46] Szeles Z., Petheő G.L., Szikora B., Kacskovics I., Geiszt M. (2023). A Novel Monoclonal Antibody Reveals the Enrichment of NADPH Oxidase 5 in Human Splenic Endothelial Cells. Sci. Rep..

[47] Kampfer C., Saller S., Windschüttl S., Berg D., Berg U., Mayerhofer A. (2014). Pigment-Epithelium Derived Factor (PEDF) and the Human Ovary: A Role in the Generation of ROS in Granulosa Cells. Life Sci..

[48] Jevitt A., Chatterjee D., Xie G., Wang X.F., Otwell T., Huang Y.C., Deng W.M. (2020). A Single-Cell Atlas of Adult *Drosophila* Ovary Identifies Transcriptional Programs and Somatic Cell Lineage Regulating Oogenesis. PLoS Biol..

[49] Piacenza L., Zeida A., Trujillo M., Radi R. (2022). The Superoxide Radical Switch in the Biology of Nitric Oxide and Peroxynitrite. Physiol. Rev..

[50] Mindrinos M.N., Petri W.H., Galanopoulos V.K., Lombard M.F., Margaritis L.H. (1980). Crosslinking of the *Drosophila* Chorion Involves a Peroxidase. Wilhelm Roux’s Arch. Dev. Biol..

[51] Konstandi O.A., Papassideri I.S., Stravopodis D.J., Kenoutis C.A., Hasan Z., Katsorchis T., Wever R., Margaritis L.H. (2005). The Enzymatic Component of *Drosophila melanogaster* Chorion Is the Pxd Peroxidase. Insect Biochem. Mol. Biol..

[52] Keramaris K.E., Konstantopoulos K., Margaritis L.H., Velentzas A.D., Papassideri I.S., Stravopodis D.J. (2020). Exploitation of *Drosophila* Choriogenesis Process as a Model Cellular System for Assessment of Compound Toxicity: The Phloroglucinol Paradigm. Sci. Rep..

[53] Margaritis L.H., Kafatos F.C., Petri W.H. (1980). The Eggshell of *Drosophila melanogaster* I. Fine Structure of the Layers and Regions of the Wild Type Eggshell. J. Cell Sci..

[54] Margaritis L.H. (1985). The Eggshell of *Drosophila melanogaster* III. Covalent Crosslinking of the Chorion Proteins Involves Endogenous Hydrogen Peroxide. Tissue Cell.

[55] Keramaris K.E., Margaritis L.H., Zografou E.N., Tsiropoulos G.J. (1996). Egg Laying Suppression in *Drosophila melanogaster* (Diptera: Drosophilidae) and Dacus (Bactrocera) Oleae (Diptera: Tephritidae) by Phloroglucinol, a Peroxidase Inhibitor. Bull. Entomol. Res..

[56] Suzuki T., Sugino N., Fukaya T., Sugiyama S., Uda T., Takaya R., Yajima A., Sasano H. (1999). Superoxide Dismutase in Normal Cycling Human Ovaries: Immunohistochemical Localization and Characterization. Fertil. Steril..

[57] Combelles C.M.H., Holick E.A., Paolella L.J., Walker D.C., Wu Q. (2010). Profiling of Superoxide Dismutase Isoenzymes in Compartments of the Developing Bovine Antral Follicles. Reproduction.

[58] Sah S.K., Agrahari G., Kim T.Y. (2020). Insights into Superoxide Dismutase 3 in Regulating Biological and Functional Properties of Mesenchymal Stem Cells. Cell Biosci..

[59] Stralin P., Karlsson K., Johansson B.O., Marklund S.L. (1995). The Interstitium of the Human Arterial Wall Contains Very Large Amounts of Extracellular Superoxide Dismutase. Arterioscler. Thromb. Vasc. Biol..

[60] Hudson A.M., Petrella L.N., Tanaka A.J., Cooley L. (2008). Mononuclear Muscle Cells in *Drosophila* Ovaries Revealed by GFP Protein Traps. Dev. Biol..

[61] Deshpande S.A., Rohrbach E.W., Asuncion J.D., Harrigan J., Eamani A., Schlingmann E.H., Suto D.J., Lee P.T., Schweizer F.E., Bellen H.J. (2022). Regulation of *Drosophila* Oviduct Muscle Contractility by Octopamine. iScience.

[62] Deady L.D., Sun J. (2015). A Follicle Rupture Assay Reveals an Essential Role for Follicular Adrenergic Signaling in *Drosophila* Ovulation. PLoS Genet..

[63] Deady L.D., Shen W., Mosure S.A., Spradling A.C., Sun J. (2015). Matrix Metalloproteinase 2 Is Required for Ovulation and Corpus Luteum Formation in Drosophila. PLoS Genet..

[64] Montezano A.C., Camargo L.D.L., Persson P., Rios F.J., Harvey A.P., Anagnostopoulou A., Palacios R., Gandara A.C.P., Alves-Lopes R., Neves K.B. (2018). NADPH Oxidase 5 Is a Pro-Contractile Nox Isoform and a Point of Cross-Talk for Calcium and Redox Signaling-Implications in Vascular Function. J. Am. Heart Assoc..

[65] Budani M.C., Tiboni G.M. (2021). Novel Insights on the Role of Nitric Oxide in the Ovary: A Review of the Literature. Int. J. Environ. Res. Public. Health.

[66] Zhang W., Chen S., Guo L., Zhang Z., Zhang J., Wang X., Meng X., Zhang M., Zhang K., Chen L. (2023). Nitric Oxide Synthase and Its Function in Animal Reproduction: An Update. Front. Physiol..

[67] Ekerhovd E., Brännström M., Alexandersson M. (1997). Evidence for Nitric Oxide Mediation of Contractile Activity in Isolated Strips of the Human Fallopian Tube A. Hum. Reprod..

[68] Berisha B., Schams D., Sinowatz F., Rodler D., Pfaffl M.W. (2020). Hypoxia-Inducible Factor-1alpha and Nitric Oxide Synthases in Bovine Follicles Close to Ovulation and Early Luteal Angiogenesis. Reprod. Domest. Anim..

[69] Fattman C.L., Schaefer L.M., Oury T.D. (2003). Extracellular Superoxide Dismutase in Biology and Medicine. Free Radic. Biol. Med..

[70] Lee P.-T., Zirin J., Kanca O., Lin W.-W., Schulze K.L., Li-Kroeger D., Tao R., Devereaux C., Hu Y., Chung V. (2018). A Gene-Specific T2A-GAL4 Library for *Drosophila*. eLife.

[71] Beauchamp C., Fridovich I. (1971). Superoxide Dismutase: Improved Assays and an Assay Applicable to Acrylamide Gels1. Anal. Biochem..

