# Supplementary material for: Orthologs of NOX5 and EC-SOD/SOD3: dNox and dSod3 Impact Egg Hardening Process and Egg Laying in Reproductive Function of Drosophila melanogaster
Source: Int J Mol Sci. 2024 Jun 2;25(11):6138. doi: 10.3390/ijms25116138 (PMC11173305; doi:10.3390/ijms25116138)
Supplement: Supplementary file 1 [file ijms-25-06138-s001.zip › ijms-2963294-supplementary.pdf]

International Journal of *Molecular Sciences*

MDPI

Article

Orthologs of NOX5 and EC-SOD/SOD3: dNox and dSod3 impact egg hardening process and egg laying in reproductive function of *Drosophila melanogaster*

Eva Louise Steinmetz [1,*], Annika Scherer [1], Célestine Calvet [1] and Uli Müller [1]

[1] Zoology & Physiology, ZHMB (Center of Human and Molecular Biology), Saarland University, Building B2.1, D-66123 Saarbrücken, Germany

* Correspondence eva.steinmetz@uni-saarland.de; Tel.: +49-681-302-6654

Supplementary figures

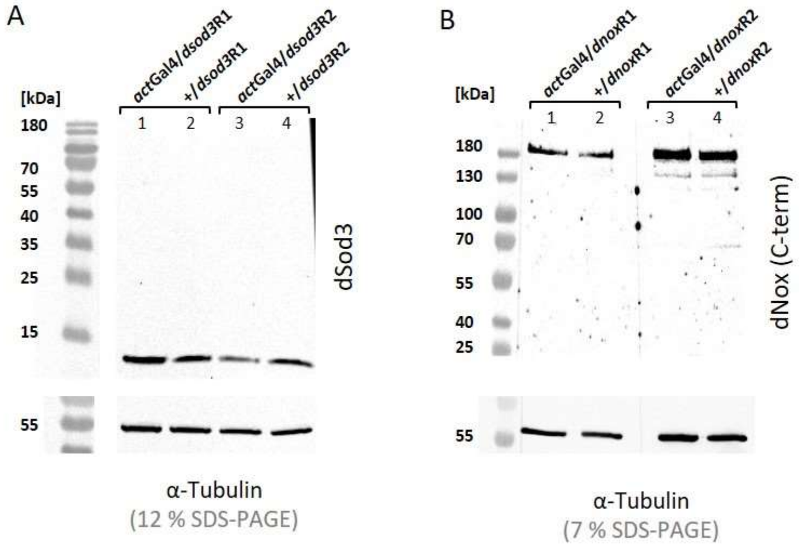

Figure S1: Western blot experiments with ovarian lysates testing dNox and dSod3 antibodies

A Ovarian lysates with RNAi downregulated *dsod*3 (*act*Gal4/*dsod*3R1; lane 1 and *act*Gal4/*dsod*3R2; lane 3) compared to control lysates without RNAi downregulation (+/*dsod*3R1; lane 2 and +/*dsod*3R2; lane 4) analyzed with anti-dSod3 antibody (1:500; #PA5-102904 Invitrogen) **B** Ovarian lysates with RNAi downregulated *dnox* (*act*Gal4/*dnox*R1; lane 1 and *act*Gal4/*dnox*R2; lane 3) compared to control lysates without RNAi downregulation (+/*dnox*R1; lane 2 and +/*dnox*R2; lane 4) analyzed with anti-dSod3 antibody (1:500; this work).

Ovarian lysates from few day old *act*Gal4/UAS-RNAi females and corresponding controls from +/UAS-RNAi females have been analyzed in western blots. We recognized a weaker band (Fig. S1, A, lane 3), when *act*Gal4/*dsod*3R2 was used to knock down *dsod*3 expression, in comparison to the control (Fig. S1, A, lane 4).

As we rechecked the target regions of the used UAS-*dsod*3RNAi lines, we found that the *dsod3*R1 line only affects *dsod*3-RE, -RF and -RD (the longer mRNA precursor (*dsod3*v2), , but not *dsod*3-RA and -RB (the shorter mRNA precursor (*dsod*3v1) (mRNA precursors of dSod3 described in [1]). In contrast, the *dsod3*R2 line targets all five transcript variants of

*dsod*3, leading to the weakened band in lane 3, caused by a more effective reduction in the total amount of dSod3. We therefore assume that the dSod3 antibody specifically recognizes the total amount of dSod3 protein, but maybe the proportion of the downregulated *dsod*3v2 is to small to be seen differentiated in lane 1.

The dNox antibody was created to recognize all four isoforms of dNox. Here too, we rechecked the target regions of the UAS *dnox*RNAi lines used (*dnox*R1 and *dnox*R2) and both lines target all transcript variants of *dnox* (-RB, -RC, -RD, -RE). The dNox protein level in *act*Gal4>*dnox*RNAi ovaries seems to be at the same level as in the control ovaries. Since we could not visualize any downregulation of the protein amount in our western blot experiments, the proof of specificity for the dNox antibody by western blot remains open.

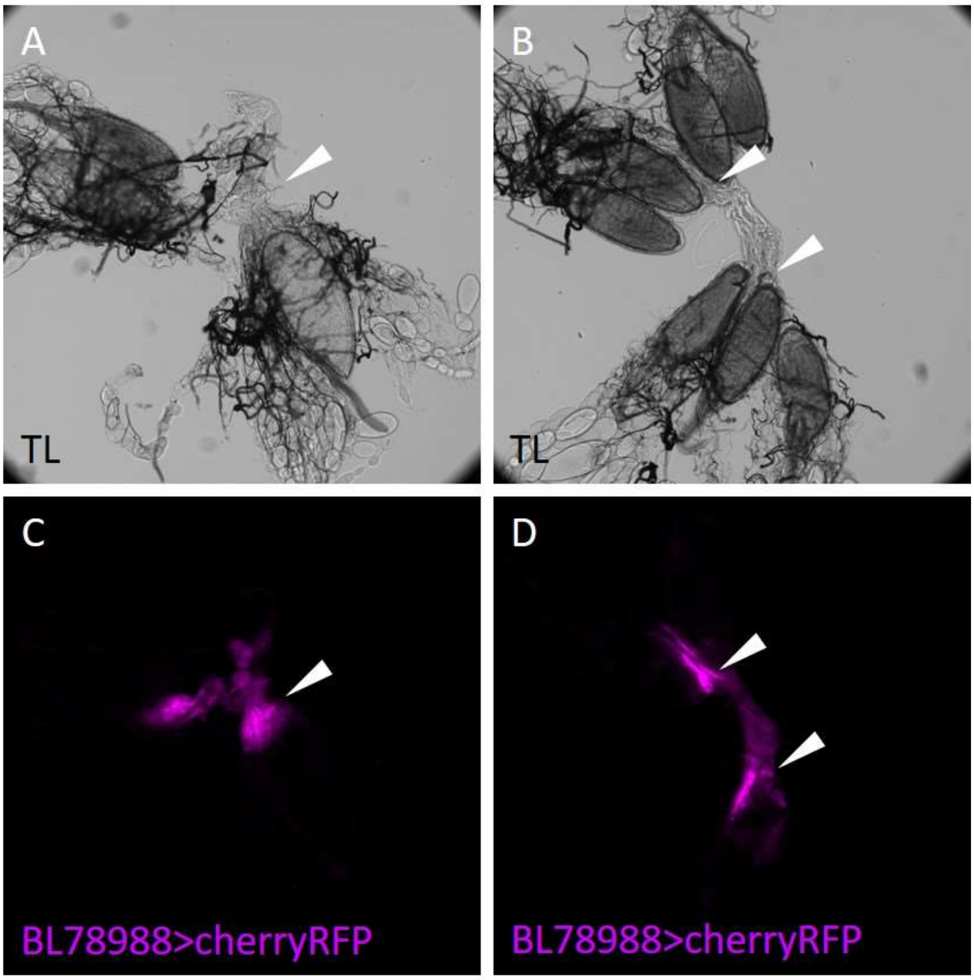

Figure S2: *dnox* expression in lateral and common oviduct of *Drosophila* ovaries
A-D ovaries expressing reporter gene (mCD8-cherryRFP) under the control of a *dnox* gene trap driver line (BL78988)

Using UAS-mCD8-cherryRFP (BL27392) reporter gene expression driven by a Gal4 driver line (BL78988) from the T2A-Gal4 library [2], we visualized *dnox* expression in lateral and common oviduct.

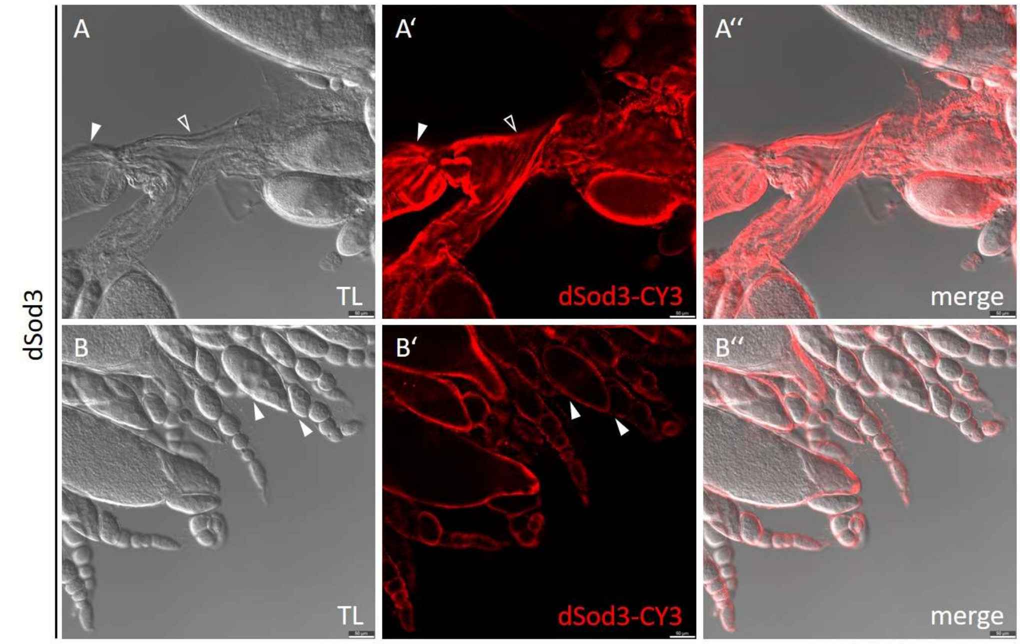

Figure S3: dSod3 localization in oviduct and ovariole muscle sheath

A-A'' lateral (white arrowhead) and common (unfilled white arrowhead) oviduct, **B-B''** ovarioles with early-stage egg chambers, surrounded by epithelial muscle sheath (white arrowheads). Fluorescent images are accompanied by the respective transmitted light (TL) images for a better identification of all structures. Indirect immunostaining was done using anti-dSod3 (1:250; #PA5-102904 Invitrogen) antibody combined with fluorophore coupled (Cy3) secondary antibodies on fixed ovaries. Images were taken with Thunder imaging system (Leica).

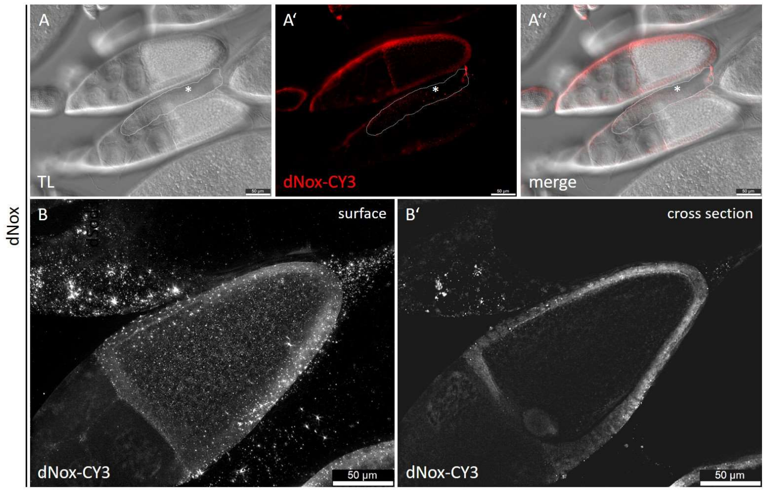

Figure S4: dNox localization in corpus luteum (CL) and follicle cells of mid-stage egg chamber

A-A'' mid-stage egg chambers and corpus luteum (CL; white asterisk) **B, B'** stage 10 egg chamber, in B focus was set to the follicle cell epithel that surrounds the growing oocyte, in B' focus was set to the cross-section level of the egg chamber for a better view at the follicle cell epithel surrounding the oocyte. Scale bars indicate 50 µm. Fluorescent images are accompanied by the respective transmitted light (TL) images for a better identification of all structures. Indirect immunostaining was

done using anti-dNox (1:250; this work) antibody combined with fluorophore coupled (Cy3) secondary antibodies on fixed ovaries. Images were taken with Thunder imaging system (Leica).

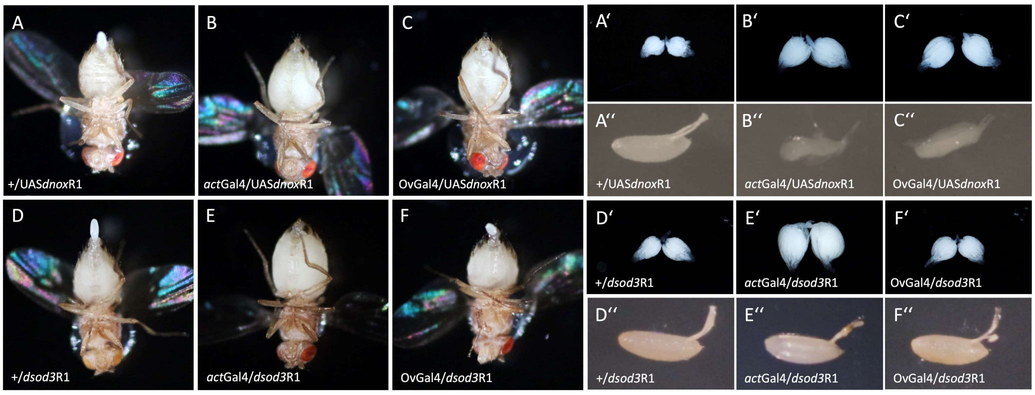

Figure S5: *dnox* RNAi knockdown with alternative effector lines *dnox*R1 (BL32433) or *dsod3*R1 (BL8760) (Extension to Fig.6 and Fig.7 in the main document; explanation is given there)

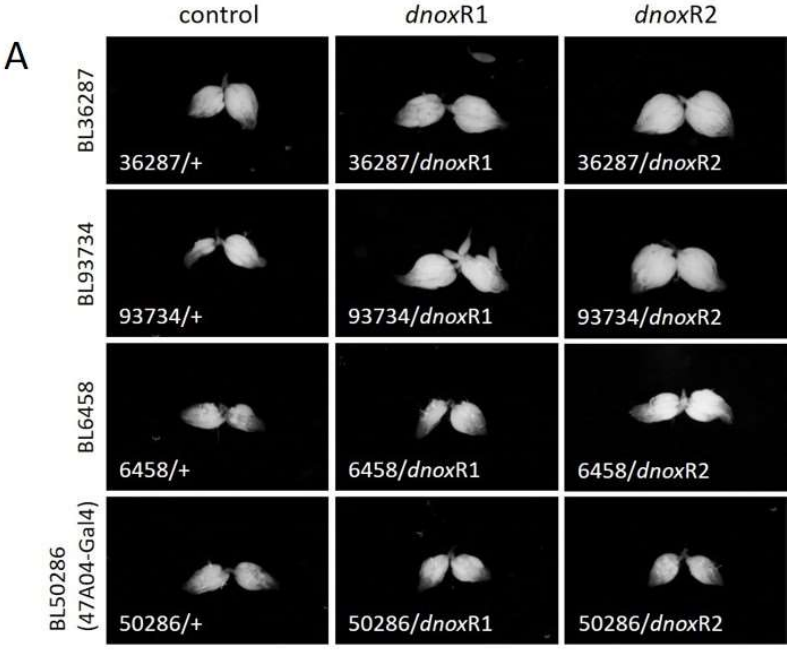

B

driver line	progeny after F1 female backcrossed?				
	control	BL32433 UAS-*dnox* R1	BL32902 UAS-*dnox* R2	BL8760 UAS-*dsod3*R1	BL37793 UAS-*dsod3* R2
BL3954 (*act* Gal4)	yes	no	no	yes	yes
BL3750 (OvGal4)	yes	no	no	yes	yes
BL36287 (GR1Gal4)	yes	no	no	yes	yes
BL93734 (CY2Gal4)	yes	no	no	yes	yes
BL6458 (slboGal4)	yes	yes	yes	yes	yes
BL50286 (47A04Gal4)	yes	yes	yes	yes	yes

Figure S6: *dnox* RNAi knockdown with alternative driver lines A ovary morphology after *dnox* RNAi knockdown; driver lines (left panel) were crossed to control line (w^{1118}) or UAS-RNAi lines *dnox*R1 or *dnox*R2 (upper panel); ovaries of few days old resulting F1 females were dissected and morphologically compared **B** fecundity test of F1-females; driver lines (left panel) were crossed to

control line (w^{1118}) or UAS-RNAi lines *dnox*R1, *dnox*R2, *dsod3*R1 or *dsod3*R2 (upper panel); resulting F1 females were backcrossed with control line males and checked if they deliver F2 individuals or not ("yes" or "no")

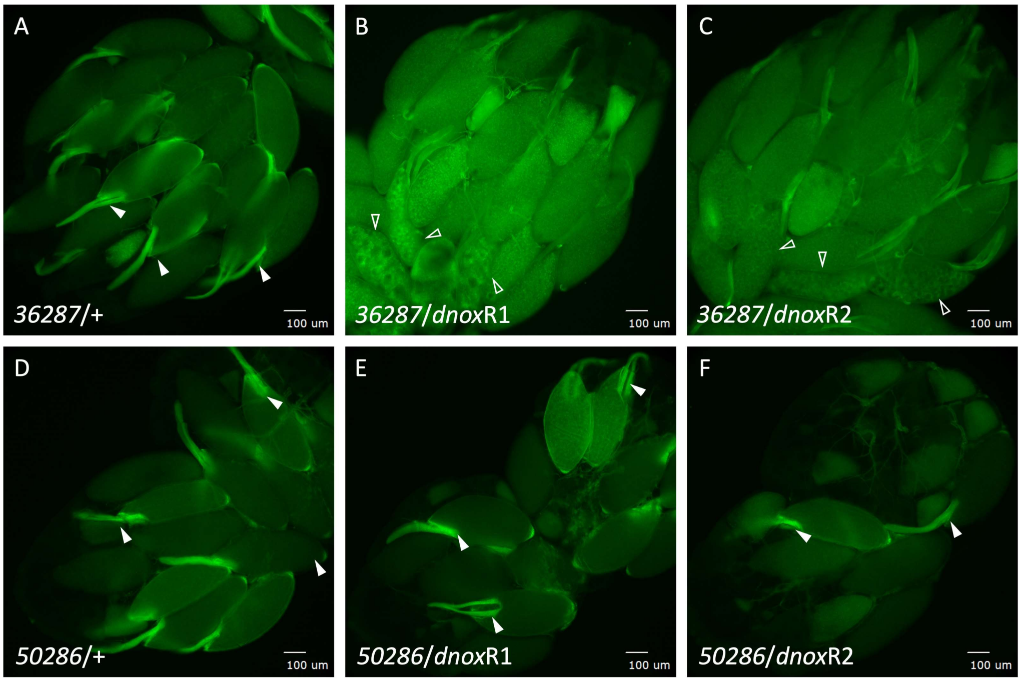

Figure S7: Chorion dysmorphology in *dnox* downregulated ovaries using alternative effector lines

A-C driver line BL36287 (GR1-Gal4) **B-F** driver line BL50286 (47A04Gal4). White arrowheads point to dorsal appendages of the chorion, unfilled white arrowheads point to the impacted yolk structure of mature egg chambers after *dnox* knockdown. Scale bars 100 µm. Excitation at 470 nm, GFP-specific emission filter.

We used alternative ovarian specific driver lines to test if they induce chorionic dysmorphologies. *dnox* knockdown by BL36287 (GR1-Gal4) (Fig.S7, B, C) revealed differences in intrinsic fluorescence compared to control (without RNAi induction) (Fig.S7, A). Furthermore, structural differences of the yolk can be seen. In contrast, *dnox* knockdown using driver line BL50286 (47A04Gal4) (Fig.S7, E, F), which was used in previous studies of [3], showed no differences of the intrinsic fluorescence or the yolk structure compared to control (Fig.S7, D).

1. Blackney, M.J.; Cox, R.; Shepherd, D.; Parker, J.D. Cloning and Expression Analysis of Drosophila Extracellular Cu Zn Superoxide Dismutase. *Biosci Rep* **2014**, *34*, 851–863, doi:10.1042/BSR20140133.
2. Lee, P.-T.; Zirin, J.; Kanca, O.; Lin, W.-W.; Schulze, K.L.; Li-Kroeger, D.; Tao, R.; Devereaux, C.; Hu, Y.; Chung, V.; et al. A Gene-Specific T2A-GAL4 Library for Drosophila. **2018**, doi:10.7554/eLife.35574.001.
3. Li, W.; Young, J.F.; Sun, J. NADPH Oxidase-Generated Reactive Oxygen Species in Mature Follicles Are Essential for Drosophila Ovulation. *Proc Natl Acad Sci U S A* **2018**, *115*, 776–7770, doi:10.1073/pnas.1800115115.
